# Genome-wide identification of polyamine metabolism and ethylene synthesis genes in *Chenopodium quinoa* Willd. and their responses to low-temperature stress

**DOI:** 10.1186/s12864-024-10265-7

**Published:** 2024-04-16

**Authors:** Xiaoxue Zhao, Shiyu Wang, Fenggen Guo, Pan Xia

**Affiliations:** 1 Faculty of Animal Science and Technology, Yunnan Agricultural University, 650201 Kunming, China; 2 College of Horticulture and Landscape, Yunnan Agricultural University, 650201 Kunming, China; 3 College of Agronomy and Biotechnology, Yunnan Agricultural University, 650201 Kunming, China

**Keywords:** *Chenopodium quinoa* Willd., Polyamine biosynthesis, Polyamine catabolism, Ethylene synthesis, Gene family analysis, Low-temperature stress

## Abstract

**Background:**

Quinoa (*Chenopodium quinoa* Willd.) is valued for its nutritional richness. However, pre-harvest sprouting poses a significant threat to yield and grain quality. This study aims to enhance our understanding of pre-harvest sprouting mitigation strategies, specifically through delayed sowing and avoiding rainy seasons during quinoa maturation. The overarching goal is to identify cold-resistant varieties and unravel the molecular mechanisms behind the low-temperature response of quinoa. We employed bioinformatics and genomics tools for a comprehensive genome-wide analysis of polyamines (PAs) and ethylene synthesis gene families in quinoa under low-temperature stress.

**Results:**

This involved the identification of 37 PA biosynthesis and 30 PA catabolism genes, alongside 227 ethylene synthesis. Structural and phylogenetic analyses showcased conserved patterns, and subcellular localization predictions indicated diverse cellular distributions. The results indicate that the PA metabolism of quinoa is closely linked to ethylene synthesis, with multiple genes showing an upregulation in response to cold stress. However, differential expression within gene families suggests a nuanced regulatory network.

**Conclusions:**

Overall, this study contributes valuable insights for the functional characterization of the PA metabolism and ethylene synthesis of quinoa, which emphasize their roles in plant low-temperature tolerance and providing a foundation for future research in this domain.

**Supplementary Information:**

The online version contains supplementary material available at 10.1186/s12864-024-10265-7.

## Background

Quinoa (*Chenopodium quinoa* Willd.) is an annual dicotyledonous herb of the family Amaranthaceae, cultivated for over 5,000 years [1]. Quinoa seeds are rich in protein and known as the “mother of grain.” By-products produced during seed production and processing, such as straw and bran, can be used as animal feed. Moreover, the leaves contain high levels of nutrients, such as protein, potassium, manganese, and copper, and can be consumed as a vegetable [2, 3].

In recent years, quinoa has become increasingly popular among the public, and the planting area has become increasingly extensive; however, the pre-harvest sprouting (PHS) has seriously affected the yield and quality of quinoa seeds [4, 5]. Appropriate delay in sowing for quinoa maturity to avoid the rainy season is a novel idea in reducing the quinoa PHS. However, certain high-altitude areas such as Shangri-La and other places in August begin to decline in temperature. Further, the nighttime temperatures can be reduced to < 0 ℃. Cultivating cold-resistant quinoa varieties is necessary to ensure that plants grow and develop normally under cold conditions. Additionally, many regions do not plant crops in winter, and quinoa, which is more resistant to cold, can utilize many winter fields. Harvesting nutrient biomass to feed livestock can also increase the income of farmers, even if it is not intended for seed harvesting. Therefore, studying the molecular mechanism of the low-temperature response in quinoa can provide genetic resources for cold-resistant breeding and theoretical guidance for popularizing quinoa cultivation.

**Fig. 1:**
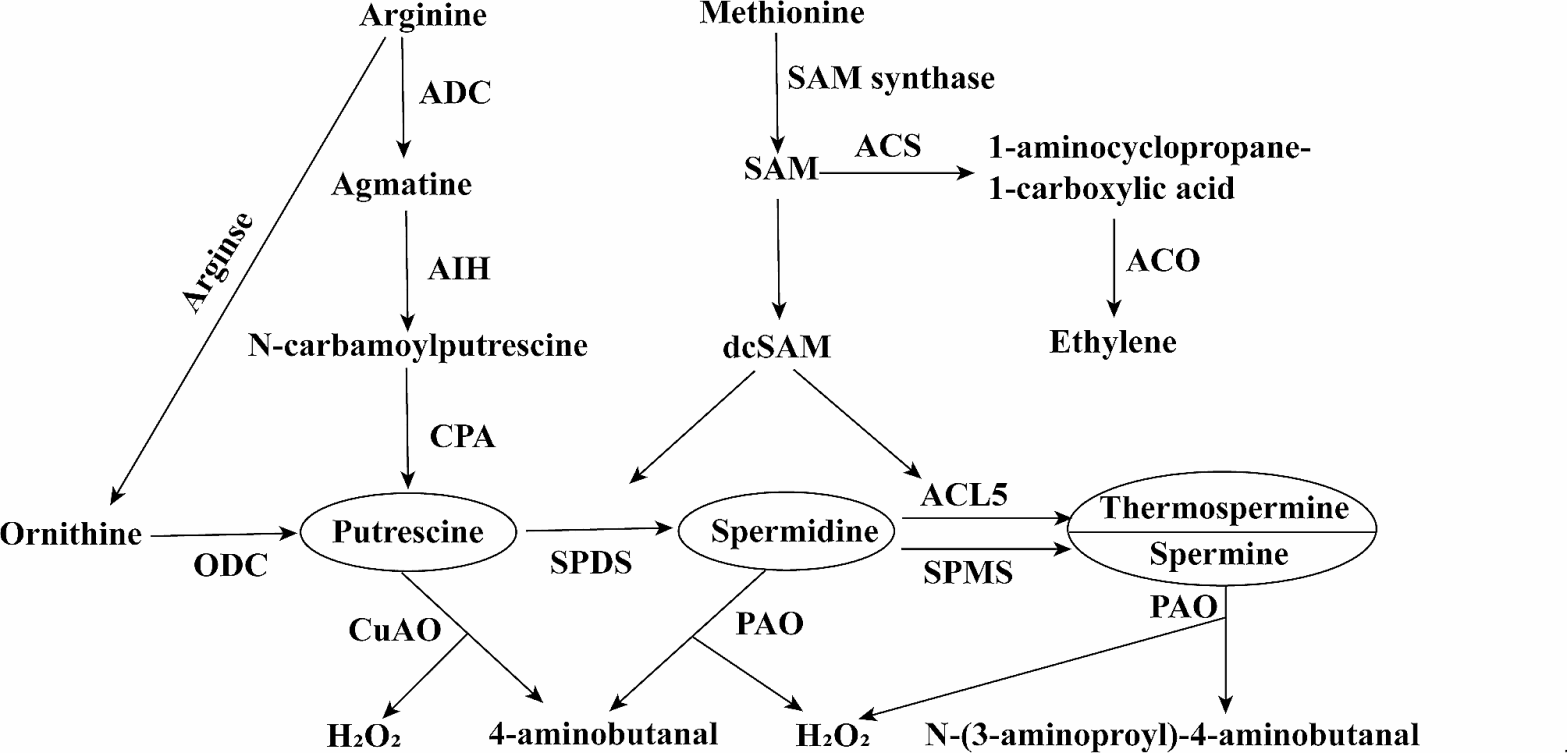
PA metabolism and ethylene synthesis pathway

Under low-temperature stress, plants experience disturbances in physiological processes including osmotic balance, photosynthetic carbon fixation, and lipid metabolism. When faced with adversity, plants activate defense mechanisms and generate responses to adapt to their environment. Polyamines (PAs), which are closely related to plant resistance, growth, and development, their levels increase in plants during abiotic stress such as salinity, extreme temperature, paraquat or heavy metals, particularly important for adaptation and resistance to cold stress, serving as crucial metabolites for responding to external stimuli [6, 7], and are a class of low-molecular-weight, aliphatic, nitrogen-containing bases with strong bioactivities that are widely present in prokaryotic and eukaryotic organisms, mainly including putrescine (Put), spermidine (Spd), spermine (Spm), thermospermine (Tspm) [8]. Under physiological pH conditions(7.0 ∼ 7.4.), PA carries a positive charge in plants and interacts with large biomolecules such as nucleic acids, proteins (enzymes), and phospholipids through hydrogen and macromolecular bonds, participating in various life processes, including the regulation of gene translation, expression, cell proliferation, and cell signal transduction, which are closely related to plant growth and development [9, 10]. They can also regulate the activity of certain ion channels, directly or indirectly participating in reactive oxygen species (ROS) metabolism in plants, act as signaling molecules in plant stress responses, and initiate the expression of resistance genes, such as those participating in responses to abiotic stresses including drought, cold, and salinity, which play an important regulatory role in plant resistance [11–13]. Two main pathways are known for PA synthesis in plant cells, the arginine decarboxylase pathway and the ornithine decarboxylase pathway, both use l-arginine as a substrate [14], as shown in Fig. 1 [15].

Arginine is converted to agmatine by the action of arginine decarboxylase (ADC, EC 4.1.1.19), which is then converted to N-carbamoylputrescine by the action of agmatine iminohydrolase (AIH, EC 3.5.3.12), followed by the catalysis of N-carbamoylputrescine amidohydrolase (CPA, EC 3.5.1.53) to form Put [15]. The second pathway involves the hydrolysis of arginine to remove urea, generating ornithine, which is then converted into Put via ornithine decarboxylase (ODC, EC 4.1.1.17) catalysis [16]. As a precursor for synthesizing other PAs, Put combines with an aminopropyl group catalyzed by spermidine synthase (SPDS, EC 2.5.1.16) to produce Spd. Spd is catalyzed by spermine synthase (SPMS, EC 2.5.1.22), thermospermine synthase (ACL5, EC 2.5.1.79), and an aminopropyl group to form Spm and Tspm, respectively.

As important plant growth regulators, the dynamic equilibrium of PAs related to their synthesis and catabolism. The catabolism of PAs is mainly catalyzed by copper-containing amine oxidases (CuAO, EC 1.4.3.6) and flavin-containing polyamine oxidases (PAO, EC1.5.3.17) [17]. Put is the central product of the PA biosynthesis pathway, and CuAO has a high affinity for Put, which is oxidatively deaminated to form H_2_O_2_, ammonia, and 4-aminobutanal catalyzed by this enzyme [17, 18]. PAO can be divided into two categories. The first group catalyzes Spd and Spm to produce 1,3-diaminopropane, H_2_O_2_ and N-(3-aminopropyl)-4-aminobutanal or 4-aminobutanal, which are involved in terminal catabolic (TC) pathways. The second type participates in the back conversion (BC) pathway, which converts Spm back to Spd, and Spd back to Put [18].

The function of PAs are largely realized through their synthesis and catabolism, and the in vivo balance of PAs is crucial [19]. Under adversity stress, some content or morphological changes in PA levels occur in plants, and to a certain extent, the strengthening of plant resistance to adversity is synchronized with the accumulation of PAs. In addition, the metabolites produced by PA decomposition play an important role in plant growth and development, stress tolerance, and defense. For example, H_2_O_2_ can be used as a signaling molecule to induce plant resistance, enhance the ability of the plant reactive oxygen species scavenging system, regulate the balance of reactive oxygen species metabolism, maintain the stable membrane structure of the cell, and is also a good mechanism to respond to adverse stress [20].

In the PA metabolic pathway, the aminopropyl group required for the synthesis of Spd, Spm, and Tspm is formed by S-adenosylmethionine decarboxylase (SAMDC, EC 4.1.1.50) catalyzed decomposition of S-adenosylmethionine (SAM) [19]. SAM is a ubiquitous methyl donor that is converted to 1-aminocyclopropane-1-carboxylic acid (ACC) under the catalysis of 1-aminocyclopropane-1-carboxylate synthase (ACS, EC 4.4.1.14), and then oxidized and decomposed by 1-aminocyclopropane-1-carboxylic acid oxidase (ACO, EC 1.14.17.4) to produce ethylene [21].

Some overlap between PA and ethylene biosynthetic pathways was observed, which may have been antagonistic. Locke et al. [22] found that using an ethylene synthesis inhibitor during barley seed germination significantly reduced the release of ethylene from barley leaves, thus promoting the synthesis of endogenous PAs in the leaves. Lasanajak et al. [23] demonstrated that during fruit ripening, an antagonistic relationship was observed between producing high-level PA and ethylene by radioactive labeling. This opposite relationship is regulated by *SAMDC* expression. Ethylene was positively correlated with senescence, whereas PAs had the opposite effect [24]. However, in transgenic tomato fruits overexpressing Yeast *SAMDC* and *SPDS*, high Spd and Spm levels were accompanied by an accumulation of *ACS* and increased ethylene content [25]. Similar changes were observed in the transgenic leaves of *Arabidopsis thaliana* overexpressing *SPMS* [26], making it challenging to establish the precise correlation between polyamines and ethylene. However, both compounds are products of plant defense response mechanisms under adverse conditions and play crucial roles in various stress responses.

Exploring PA and ethylene responses to low-temperature stress in quinoa can help to understand the mechanisms of quinoa cold resistance and enrich the theory of plant cold resistance. When studying low-temperature stress, 4 °C is a commonly used stress condition, which simulates the environment of low temperatures in early spring or late fall seasons, a temperature that can trigger a low-temperature response in the plant without causing immediate cellular frostbite, and enables researchers to observe the performance of the plant under mild to moderately intense low-temperature stresses to assess its cold tolerance [27–29]. Previously, we collected 39 quinoa resources from various regions in Yunnan Province, China, and through screening experiments, we obtained two materials with disparate cold resistance, i.e., low-temperature-tolerant red quinoa RQ8 and low-temperature-sensitive white quinoa WQ13, and determined that 48 h of stress at 4 °C was sufficient to cause quinoa to produce an obvious stress response, including physiological and molecular-level changes without causing excessive damage, which allowed us to better evaluate quinoa’s cold-tolerant and recovery abilities. Therefore, in this study, with the help of bioinformatics, we screened, identified and analyzed the PA metabolism gene family and ethylene synthesis gene family of quinoa in the quinoa genome, and analyzed the expression patterns of PA metabolism and ethylene synthesis genes in response to low-temperature stress by combining the transcriptomic and proteomic data of two quinoa materials with different cold tolerance under 48 h of 4 °C stress, in order to explore the roles of both in response to the process of cold stress.

## Materials and methods

### Identification of PA metabolism and ethylene synthesis genes in quinoa

Hidden Markov Model (HMM) files of conserved structural domains of PA biosynthesis, PA catabolism, and ethylene synthesis genes were obtained from Pfam. Quinoa genome-wide data were obtained from the National Center of Biotechnology Information database (NCBI, https://www.ncbi.nlm.nih.gov). Quinoa genome-wide protein sequences were scanned using hmmsearch and filtered by setting the e-value to < 1e^-5^. The filtered quinoa protein sequences were used to construct a new species-specific HMM and searched. Protein sequences of relevant gene family members were downloaded from the Arabidopsis (https://www.arabidopsis.org/) and rice genome databases(http://rice.uga.edu/analyses_search_blast.shtml) [17, 30–34], and E values < 1e-^10^ were set to perform blastp homology comparisons with quinoa. The Pfam search and BLASTP results were integrated, and proteins containing the target structural domains were identified as gene family members using NCBI-CDD (http://www.ncbi.nlm.nih.gov/Structure/cdd/wrpsb.cgi).

The number of amino acids, theoretical isoelectric point (pI), molecular weight (Mw), instability index(Ii), aliphatic index (Ai), and grand average of hydropathy (GRAVY) of gene families were analyzed using TB tools. Subcellular localization was predicted using the online software subCELlular LOcalization predictor ( http://cello.life.nctu.edu.tw/).

### Construction of phylogenetic tree of quinoa PA metabolism and ethylene synthesis genes

Protein sequences were compared by clustering using MEGA 11.0. The bootstrap value was set to 1,000, and the maximum likelihood method was used to construct a phylogenetic tree of the gene families of quinoa, Arabidopsis, and rice.

### Analysis of gene motif, promoter cis-acting elements, and gene structure

Protein-conserved motifs were analyzed using the MEME online tool (https://meme-suite.org/meme/tools/meme); 2,000 bp of upstream sequences of the genes were extracted using TBtools 2.031 software and submitted to PlantCare (http://bioinformatics.psb.ugent.be/webtools/plantcare/html/) to analyze the promoter cis-acting elements. The gene structures were predicted using TBtools software and the intron-exon maps of the genes were obtained, and all the above results were visualized with TBtools software.

2.4 Analysis of expression patterns of quinoa PA metabolism and ethylene synthesis gene family members under cold stress.

Red quinoa (RQ8) seeds resistant to low temperatures and white quinoa (WQ13) seeds susceptible to low temperatures were sown in pots (caliber: 100 cm; height: 85 cm; bottom diameter: 70 cm) filled with 0.8 kg nutrient-rich soil (Table S1 for nutritional components) and gently compacted. These pots were placed in an incubator, at 25 ℃/20 ℃ (14 h light/10 h dark) temperature regime, 70% relative humidity and photosynthetic photon flux density (PPFD) of 190 µmol·(m^2^·s)^-1^. When the quinoa grew to 6 true leaves, some of the plants were removed from the incubator and put into a low-temperature incubator with the same light and humidity conditions, and the temperature was adjusted to 4 ℃. 48 h later, quinoa leaves under normal and low-temperature conditions were collected for transcriptome and proteome sequencing analysis, respectively. Transcriptome and proteomic sequencing analyses were conducted, and relevant graphs were drawn using the R language. RQ8 and WQ13 in the control group were abbreviated as ZR and ZW. Red quinoa and white quinoa in the treatment group were abbreviated as LZR and LZW, respectively.

Transcriptome methods: RNA was extracted from different materials, and cDNA was synthesized using RNA as a template with a mixture of 6-base random primers and reverse transcriptase to generate the first strand cDNA. The second strand cDNA synthesis was then carried out using the first strand cDNA as a template. After library construction, Next-Generation Sequencing was performed on the library using the Illumina HiSeq sequencing platform with paired-end (PE) sequencing. The raw data was filtered to obtain high-quality clean data, which was then aligned to the reference genome of quinoa (GCF_001683475.1_ASM168347v1_genomic.fna). Gene expression levels were calculated based on the alignment results. Further differential expression analysis, enrichment analysis, and clustering analysis were conducted on the samples. Reads that aligned were assembled to reconstruct the transcript sequences.

Proteomics methods: Proteins were extracted from different materials, quantified using the BCA method, digested using FASP, and the resulting peptides were dried and reconstituted with 0.1% TFA for peptide concentration determination for LC-MS analysis. Each sample was then subjected to chromatographic separation using the Easy nLC 1200 chromatography system (Thermo Scientific). Mass spectrometry database search was conducted using MSFragger 3.4 software with the Uniprot protein database (Uniprot Chenopodium quinoa (Quinoa)[63459]-34188-202201030.fasta) for protein sequence alignment and identification.

## Results

### Identification, physicochemical properties, and phylogeny analysis of PA metabolism and ethylene synthesis gene families

An HMM matrix search was performed on quinoa genome protein sequence files, and subsequently, a BLASTP comparison was performed with Arabidopsis and rice PA metabolism proteins and ethylene synthesis proteins. After integration, 37 genes, including two *CqADCs*, two *CqODCs*, two *CqAIHs*, two *CqSPDSs*, two *CqSPMSs*, three *CqACL5s*, seven *CqSAMDCs*, and 17 *CqCPAs* were identified from the quinoa PA biosynthesis pathway. Six *CqCuAOs* and 24 *CqPAOs* in PA catabolism, 35 *CqACSs*, and 192 *CqACOs* in the ethylene synthesis pathway were identified (Table S2).

The lengths of the proteins involved in the PA synthesis pathway ranged from 80 (CqCPA16) to 747 (CqADC1) amino acids. Their Mw were 9382.99 (CqCPA16) to 80138.71 Da (CqADC1), whereas pI ranged from 4.69 (CqSAMDC7) to 9.37 (CqCPA14). Their Ii was between 25.25 (CqCPA6) to 48.13 (CqSPMS2), of which 21 proteins were unstable (Ii>40). Their Ai ranged from 74.78 (CqCPA4) to 95.14 (CqODC2), while the GRAVY ranged from − 0.429 (CqAIH2) to 0.057 (CqSAMDC6), with 81.58% hydrophilic proteins. Subcellular localization prediction showed that CqCPA14 and CqSAMDC2 were localized in the nucleus; CqCPA17, CqSAMDC3, CqSAMDC4, CqSAMDC6 and CqSAMDC7 were localized in the plasma membrane; CqADC2, CqCPA1, CqCPA2, CqCPA8 ∼ CqCPA13, CqCPA15, CqCPA16, CqSAMDC1, CqSAMDC5 in chloroplasts. All the other genes were localized in the cytoplasm.

The lengths of the PA catabolic proteins ranged from 281 (CqCuAO3) to 1,939 (CqPAO16) amino acids. Their Mw were 32,151.04 (CqCuAO3) to 210,966.23 Da (CqPAO16), while the pI ranged from 5.08 (CqPAO22) to 9.38 (CqPAO9). The Ii ranged from 30.88 (CqPAO18) to 46.76 (CqPAO1), of which 9 proteins belonged to unstable proteins. Their Ai were 75.87 (CqPAO5) to 98.72 (CqPAO18), whereas GRAVY ranged from − 0.469 (CqPAO16) to 0.009 (CqPAO18), except CqPAO18; all other proteins were hydrophilic. The predicted subcellular localization results showed that CqPAO16 and CqPAO24 were localized in the nucleus; CqCuAO1, CqCuAO4, CqCuAO5, CqPAO9, CqPAO21 in the plasma membrane; CqPAO1, CqPAO3, CqPAO7, CqPAO10, CqPAO17, CqPAO22 in the chloroplasts and cytoplasm.

The lengths of the ethylene anabolic proteins ranged from 234 (CqACO109) to 884 (CqACO166) amino acids. Their Mw were 27104.85 (CqACO109) to 100879.54 Da (CqACO166), while pI ranged from 4.82 (CqACO117) to 8.77 (CqACO79). Their Ii ranged from 17.51 (CqACO56) to 59.05 (CqACO187), while Ai were 67.33 (CqACO188) to 107.49 (CqACO117). The GRAVY were at -0.615 (CqACO96) to 0.052 (CqACS14), where CqACS4, CqACS35, CqACS4, CqACS35, CqACO117, CqACS14 were hydrophobic proteins. The predicted subcellular localization results showed that ethylene anabolic genes were mainly localized in the cytoplasm, nucleus, chloroplasts, and plasma membrane.

**Fig. 2:**
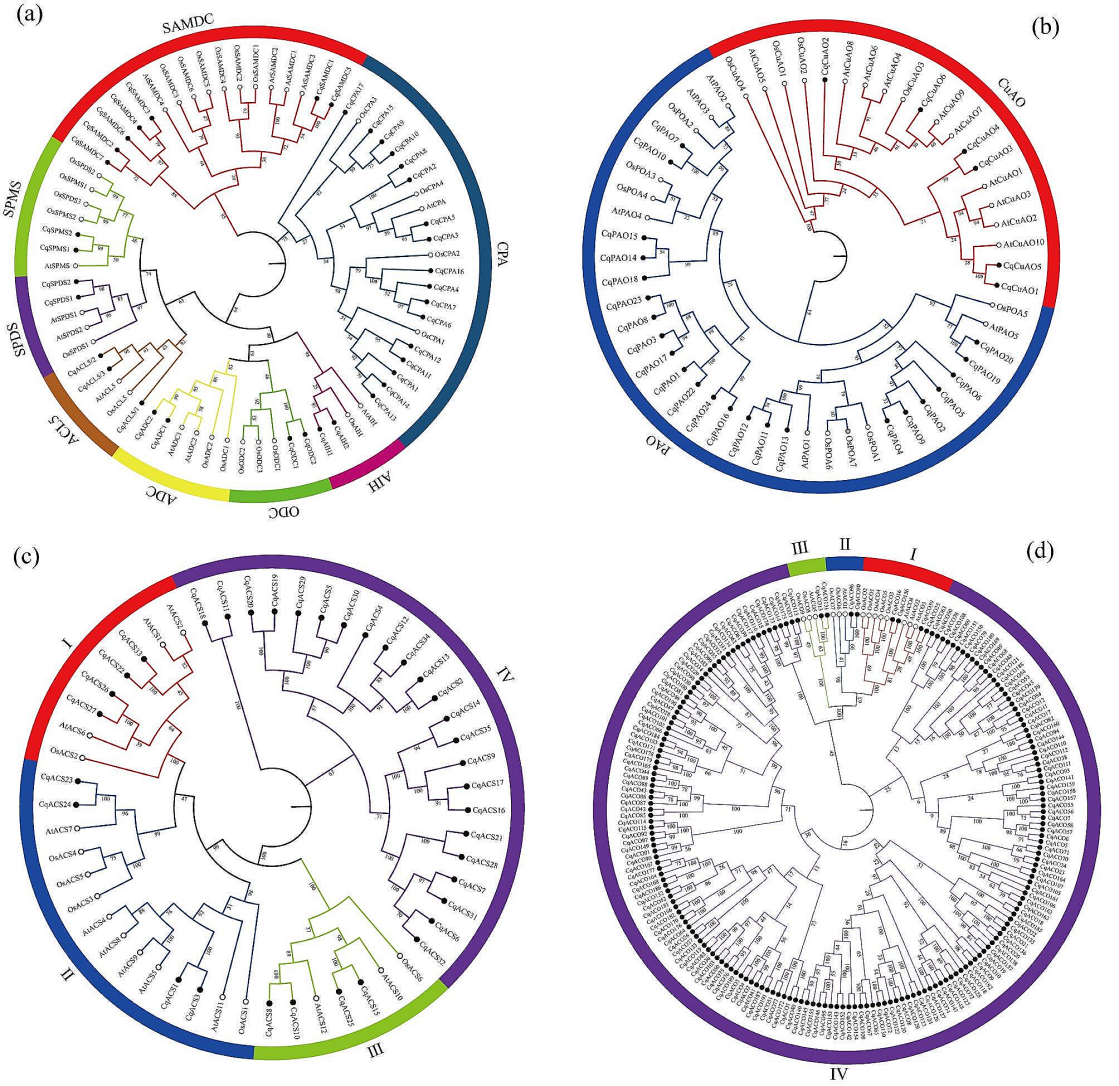
Phylogenetic trees of quinoa, Arabidopsis, and rice (**a**) PA synthesis gene family, (**b**) PA catabolism gene family, (**c**) *ACS* gene family in the ethylene synthesis pathway, (**d**) *ACO* gene family in the ethylene synthesis pathway

Phylogenetic trees of the PA metabolism and ethylene biosynthesis gene families in quinoa, Arabidopsis, and rice were constructed (Fig. 2). Fewer members of *CqADC*, *CqODC*, *CqSPDS*, *CqSPMS*, *CqAIH*, and *CqACL5* families were observed in the PA synthesis pathway. *CqADC*, *CqSPDS*, and *CqSPMS*, as well as *CqACL5/2* and *CqACL5/3*, were more closely related to Arabidopsis, whereas *CqAIH* and *CqACL5/1* were more closely related to rice. *CqODC* was only compared with *OsODC* in rice because no *ODC* family was observed in Arabidopsis. *CqSAMDC1* and *CqSAMDC5* were similar to those in Arabidopsis. More members of the *CqCPA* family were found, and most were closely related to rice.

### Analysis of conserved motif, cis-acting element of promoter and gene structure

**Fig. 3:**
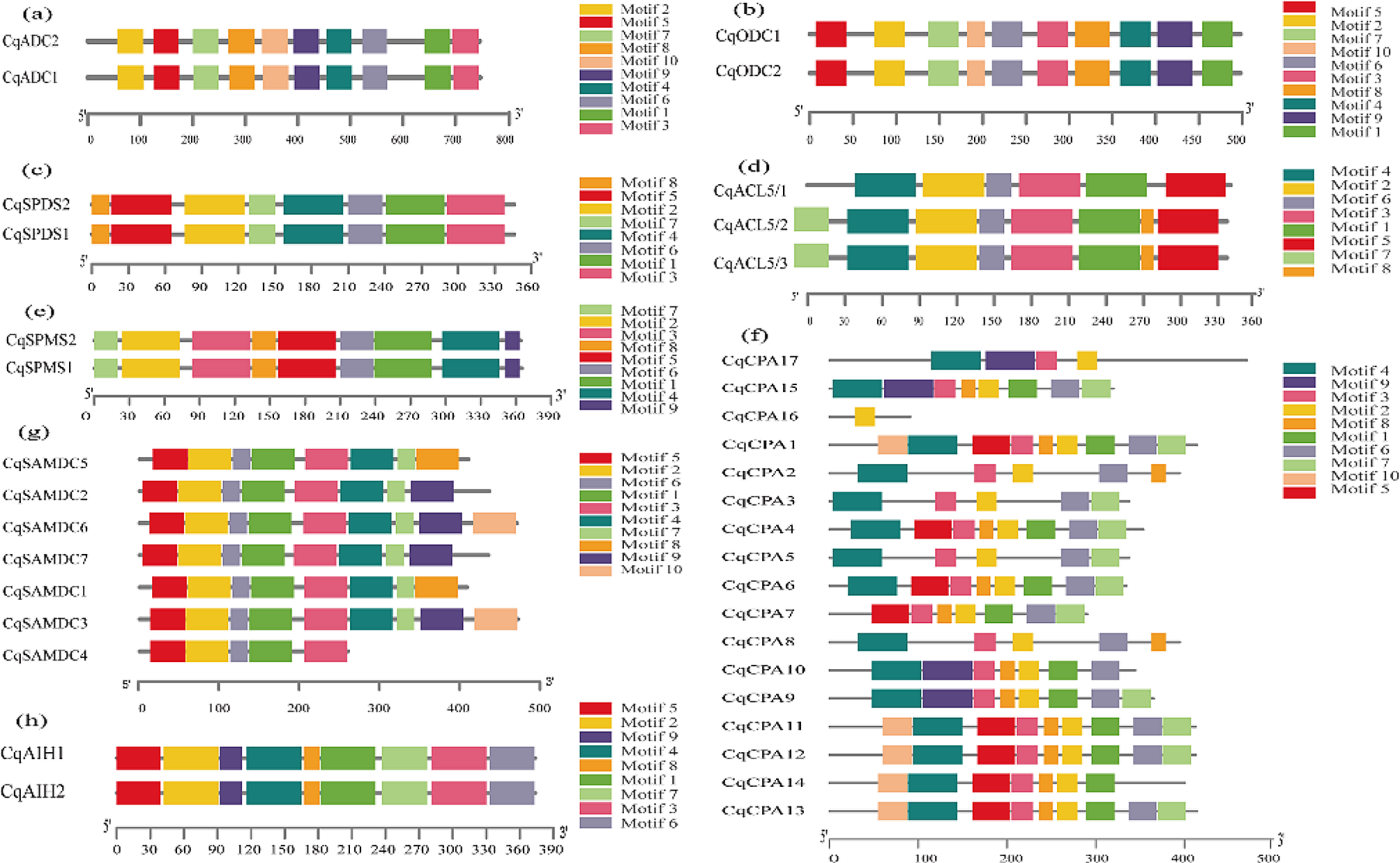
Conserved motifs of quinoa PA synthesis pathway genes (**a**) *CqADC* family, (**b**) *CqODC* family, (**c**) *CqSPDS* family, (**d**) *CqACL5* family, (**e**) *CqSPMS* family, (**f**) *CqODC* family, (**g**) *SAMDC* family, (**h**) *CqAIH* family

Members of the same family have similar conserved motifs (Fig. 3). The conserved motifs *CqADC*, *CqODC*, *CqSPDS*, *CqSPMS*, and *CqAIH* in the PA synthesis pathway were identical to those of members of the same family. All CqACL5 family members contain motifs 1 ∼ 6, and *CqACL5/2*, as well as *CqACL5/3*, contain motifs 7 and 8. In the *CqSAMDC* family, motifs 1 ∼ 3, 5, and 6 are highly conserved and present in each member. In the *CqCPA* family, motif 2 is the most conserved motif and is present in all members. Except for *CqCPA16*, all members of the *CqCPA* family contain motif 3. In the catabolism of PA (Figs. S1), motif 1, motif 5 and motif 6 were the most conservative. Excluding *CqCuAO2* and *CqCuAO3*, all other members of the *CqCuAO* family had the same motif and were arranged similarly. In the *CqPAO* family, motif 3 and motif 4 were the most conserved, followed by motif 7 and motif 9. In the ethylene synthesis pathway (Figs. S2 and S3), all members of the *CqACS* family had motif 1, motif 5, and motif 6, and all members had Motif 8 except for *CqACS21*. In the *CqACO* family, motif 4 was the most conserved, except for *CqACO124* and *CqACO161*, all other members had motif 3 and motif 6.

The exon-intron structural diversity of gene families is important for studying the evolution and function of gene family members. The results of gene structure analysis (Figs. S4 ∼ S7) showed that the structural features of quinoa PA anabolic genes showed a clear family distribution pattern. The members of the same family, possessed identical structural features, *CqADC*, *CqODC*, *CqSPDS*, *CqSPMS*, and *CqACL5* with 10, 10, 10, 10, 8, 1 exon, and 8, 10, 9, 7, 0 intron, all of them had 5’ and 3’ UTR, respectively. *CqSAMDC1*, *CqSAMDC2*, *CqSAMDC5*, and *CqSAMDC7* had the same structure, including 1exon and 2 introns, whereas *CqSAMDC3* and *CqSAMDC6* did not. The number of exons in the *CqCPA* family ranged from 3 to 10, and the number of introns ranged from 2 to 9. *CqCPA16* had a simpler structure containing 3 exons, 2 introns, and 3’ UTR, while *CqCPA4* and *CqCPA6* were structurally complex containing 10 exons, 9 introns, and 5 ’and 3’ UTR. In the catabolic pathway of PA, *CqCuAO2*, and *CqCuAO6* are similar in structure, with 5 exons and 4 introns. Excluding *CqCuAO1* and *CqCuAO5* which only had a 3’ UTR, the other family members had 5’ and 3’ UTRs. In the *CqPAO* family, *CqPAO1*, *CqPAO19* and *CqPAO22* have 1 exon and no introns. In the ethylene synthesis pathway, the number of exons in the *CqACS* family ranged from 3 to 15, with introns from 2 to 14, *CqACS23* and *CqACS24* without UTR, and *CqACS* 14 had only a 3’ UTR. The number of exons in the *CqACO* family ranged from 2 to 12, of which 88 had 3 exons and 54 had 4 exons. *CqACO5*, *CqACO6*, *CqACO55*, and *CqACO57* had the most complex structure, containing 12 exons, 11 introns, and 5’ and 3’ UTR.

### Quality testing of sequencing data

More than 94% clean reads were obtained for all 12 samples, and after purification, the Q20 base ratio was greater than 98%, and the Q30 base ratio was > 94% (Tables S3, S4). PCA can cluster similar samples together, and the closer the distance, the higher the similarity between samples. The correlation of gene expression level between samples is an important index to test the reliability of the experiment and determine whether the sample selection is reasonable. These correlations between samples were expressed by the Pearson correlation coefficient (Fig. S8); the repeatability and similarity of the LZW, LZR, ZW, and ZR samples were better, and the quality of the sequencing data was higher, which could be used for subsequent analysis.

### Upset venn diagram analysis of PA metabolism and ethylene synthesis genes

**Fig. 4:**
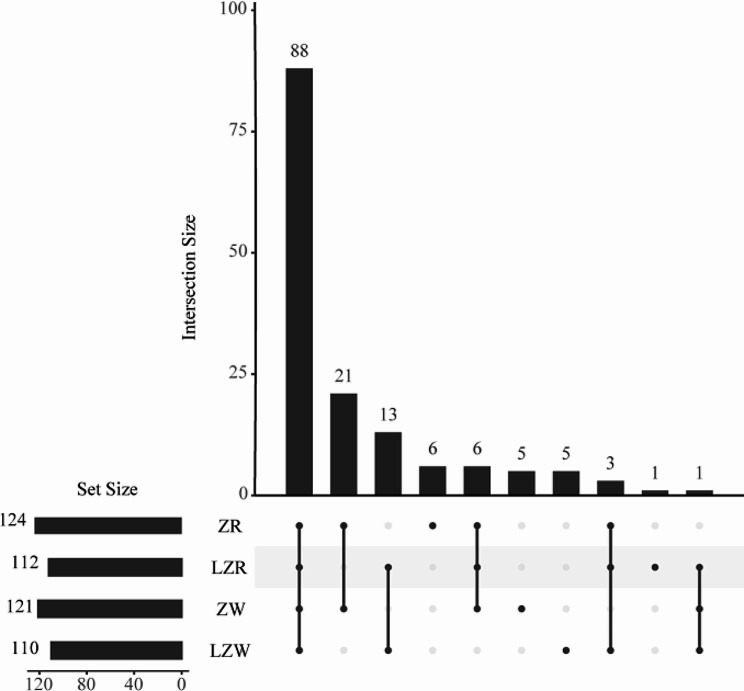
Upset analysis of PA metabolism and ethylene synthesis genes

The distribution of family genes in the samples was demonstrated using an Upset Venn diagram (Fig. 4). A total of 149 genes were detected in the transcriptomes of the PA metabolism and ethylene synthesis families, among which 88 genes were present in all four samples, 21 genes were only present in ZR and ZW, and 13 genes were only present in LZR and LZW. The genes unique to ZR, LZR, ZW, and LZW were 6, 1, 5 and 5, respectively.

### Expression of PA metabolism and ethylene synthesis genes under low temperature stress

**Fig. 5:**
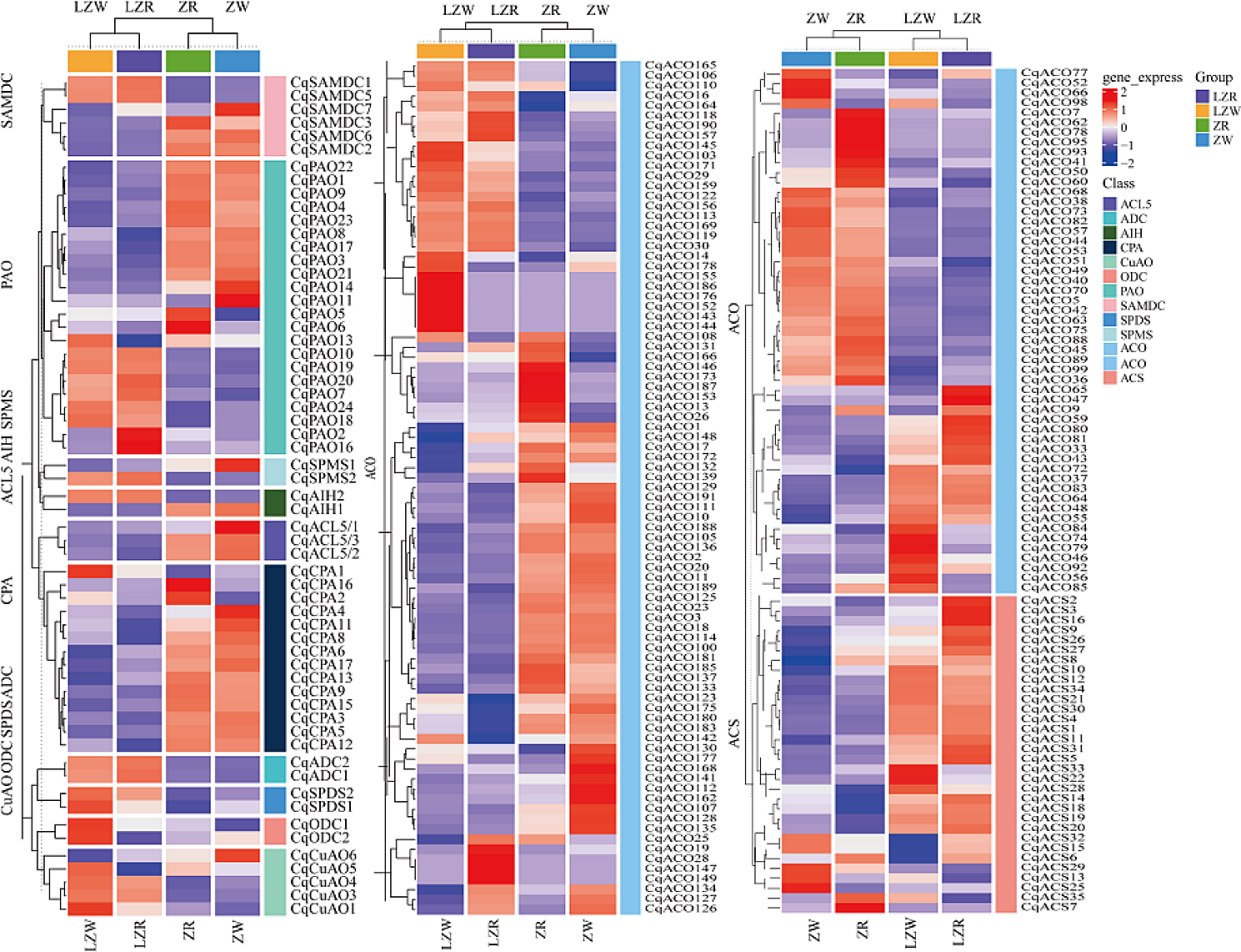
Expression changes of PA metabolism and ethylene synthesis genes under low temperature stress

Different gene families showed differential expression under cold stress, indicating specificity in the expression of these genes (Fig. 5). In the PA synthesis pathway, the *CqSPDS* and *CqADC* family genes showed a consistent up-regulation trend in expression after low-temperature stress. In contrast, the opposite trend was found for *CqACL5* and *CqCPA*. *CqODC* was highly expressed in LZW. *CqCPA* was highly expressed under non-stress conditions, whereas *CqCPA1* showed increased expression under stress. The *CqAIH* family is comprised of two genes with completely different expression patterns. *CqAIH1* was highly expressed before stress exposure, whereas the opposite was true for *CqAIH2*. A similar situation was observed for the *CqSPMS* and *CqSAMDC* families. *CqSAMDC2*, *CqSAMDC3*, and *CqSAMDC6* were highly expressed before stress, whereas the opposite was true for *CqSAMDC1* and *CqSAMDC5*. Among the PA catabolic gene families, *CqCuAO5* and *CqCuAO6* were highly expressed before stress, whereas other genes were highly expressed after stress. After low-temperature stress, the expression levels of *CqPAO7*, *CqPAO10*, *CqPAO18* ∼ *CqPAO20* and *CqPAO24* were upregulated, whereas those of *CqPAO1*, *CqPAO3*, *CqPAO4*, *CqPAO8*, *CqPAO9*, *CqPAO14*, *CqPAO17* and *CqPAO21* ∼ *CqPAO23* were decreased. In the ethylene synthesis pathway, most gene members of the *CqACS* family showed higher expression after stress. 37 genes of the *CqACO* family had upregulated expression and 51 genes showed decreased expression after low-temperature stress, whereas the other members showed no obvious expression pattern.

### Expression analyses of differential genes in PA metabolism and ethylene synthesis under low temperature stress

**Fig. 6:**
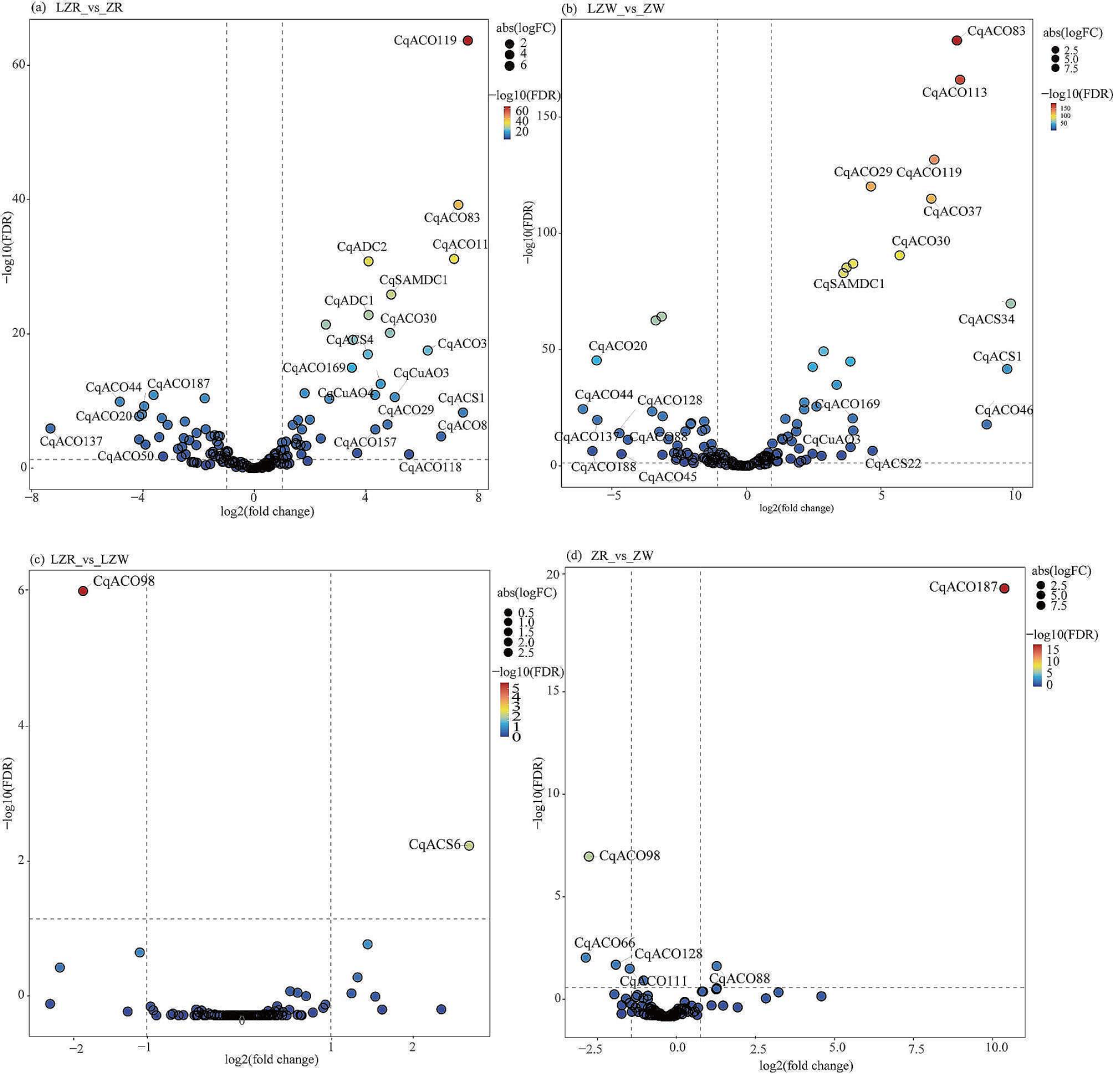
Expression of differential genes in PA metabolism and ethylene synthesis, (**a**) differential expression in LZR_vs_ZR, (**b**) differential expression in LZW_vs_ZW, (**c**) differential expression in LZR_vs_LZW, (**d**) differential expression in ZR_vs_ZW

In the LZR_vs_ZR and LZW_vs_ZW comparison groups, the number of upregulated genes was high, and the expression of *CqACO30*, *CqACO37*, *CqACO83*, *CqACO113*, *CqACO119*, *CqACO169*, *CqACS1*, *CqSAMDC1*, and *CqCuAO3* was significantly upregulated, with a fold difference of more than 10-fold ( FPKM), in which the expression levels of *CqACO83* and *CqACO119* varied more than 100-fold (FPKM), indicating a strong response to low temperature (Fig. 6); *CqACO20*, *CqACO44*, and *CqACO137* were significantly downregulated in both groups by more than 20-fold (FPKM). The number of differentially expressed genes in different materials at the same temperature was lower. *CqACO98* was significantly downregulated in LZR_vs_LZW and ZR_vs_ZW, and *CqACS6* and *CqACO187* were significantly upregulated. The expression of gene family members in the three metabolic pathways fluctuated greatly between LZR_vs_ZR and LZW_vs_ZW.

Taking LZR_vs_ZR as an example, the differentially expressed family genes were subjected to GO enrichment analysis, and GO classification was performed according to their molecular function, biological processes, and cellular components (Fig. S9). The molecular functions of the differentially expressed genes were mainly enriched in 6 areas namely 2 − oxoglutarate − dependent dioxygenase activity, dioxygenase activity, L-ascorbic acid binding, monosaccharide binding, pyridoxal phosphate binding, and vitamin binding. Biological processes were mainly enriched in amine biosynthesis, amine metabolism, biogenic amine biosynthesis, biogenic amine metabolism, polyamine biosynthesis, and polyamine degradation. The cellular components were mainly enriched in microbodies and peroxisomes.

KEGG analysis (Fig. S10, Table  S5) showed that differentially expressed genes in LZR_vs_ZR were involved in 11 pathways, including cysteine and methionine metabolism; arginine and proline metabolism; isoquinoline alkaloid biosynthesis; flavonoid biosynthesis; phenylalanine metabolism; tropane, piperidine, and pyridine alkaloid biosynthesis; tyrosine metabolism; diterpenoid biosynthesis; ubiquinone and other terpenoid-quinone biosynthesis; phenylalanine, tyrosine, and tryptophan biosynthesis; and beta-alanine. Among them, more genes were enriched in cysteine and methionine metabolism, namely *CqACO156*, *CqACS1*, *CqACS34*, *CqACS4*, *CqSAMDC1*, *CqSAMDC5*, and *CqSPMS2*, all of which were upregulated.

### Gene set enrichment analyses of genes in PA metabolism and ethylene synthesis

**Fig. 7:**
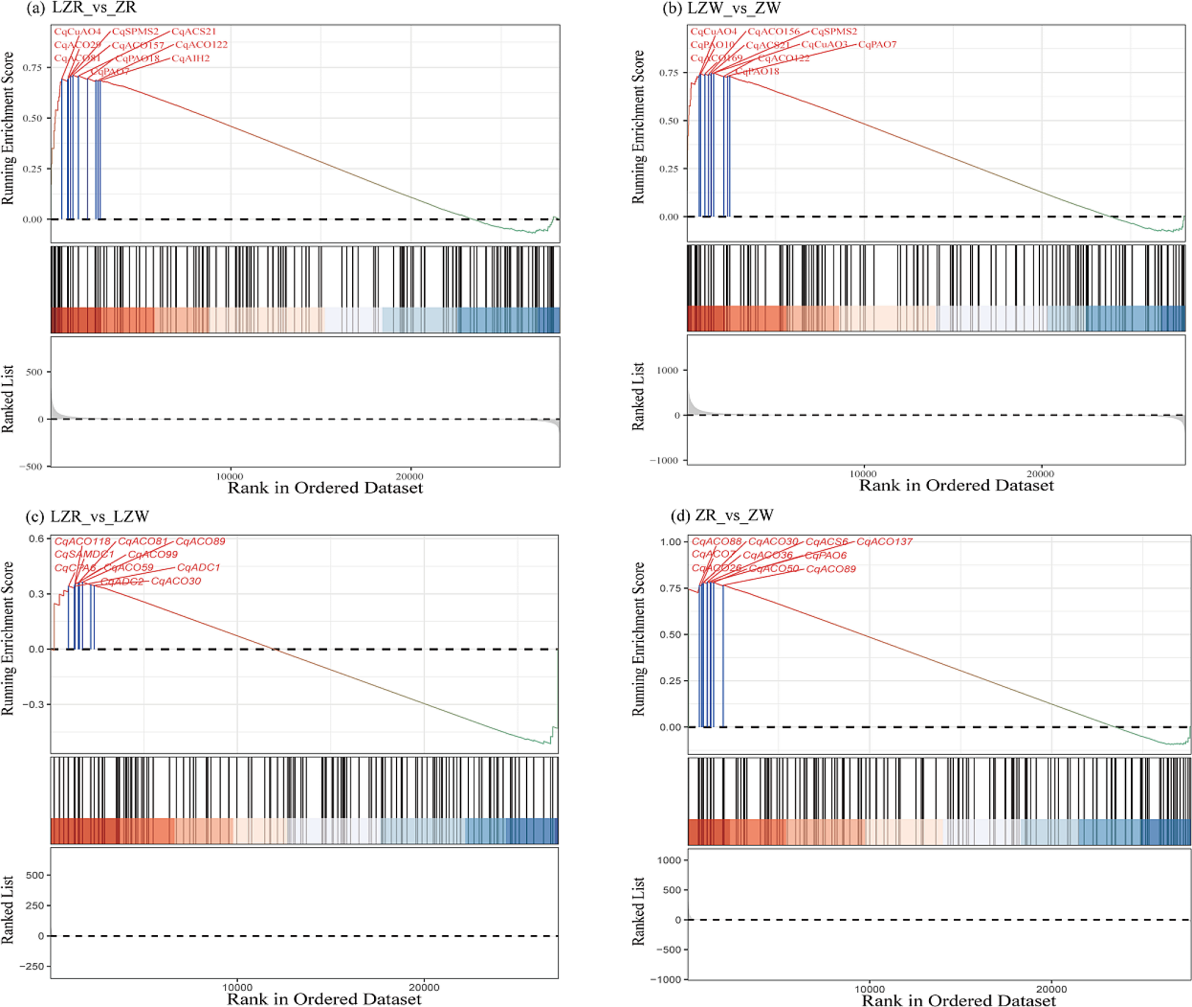
GSEA of genes in PA metabolism and ethylene synthesis

Gene set enrichment analysis (GSEA) was utilized to explore further the overall expression of the 12 gene families (Fig. 7). The families were upregulated in LZR_vs_ZR, LZW_vs_ZW, and ZR_vs_ZW, and *CqSPMS2*, *CqCuAO4*, *CqPAO7*, *CqPAO18*, and *CqACS21* accounted for important positions among the upregulated genes. In LZR_vs_LZW, the proportions of upregulated and downregulated genes were approximately the same.

### Protein-protein interaction analyses of PA metabolism and ethylene synthesis genes

**Fig. 8:**
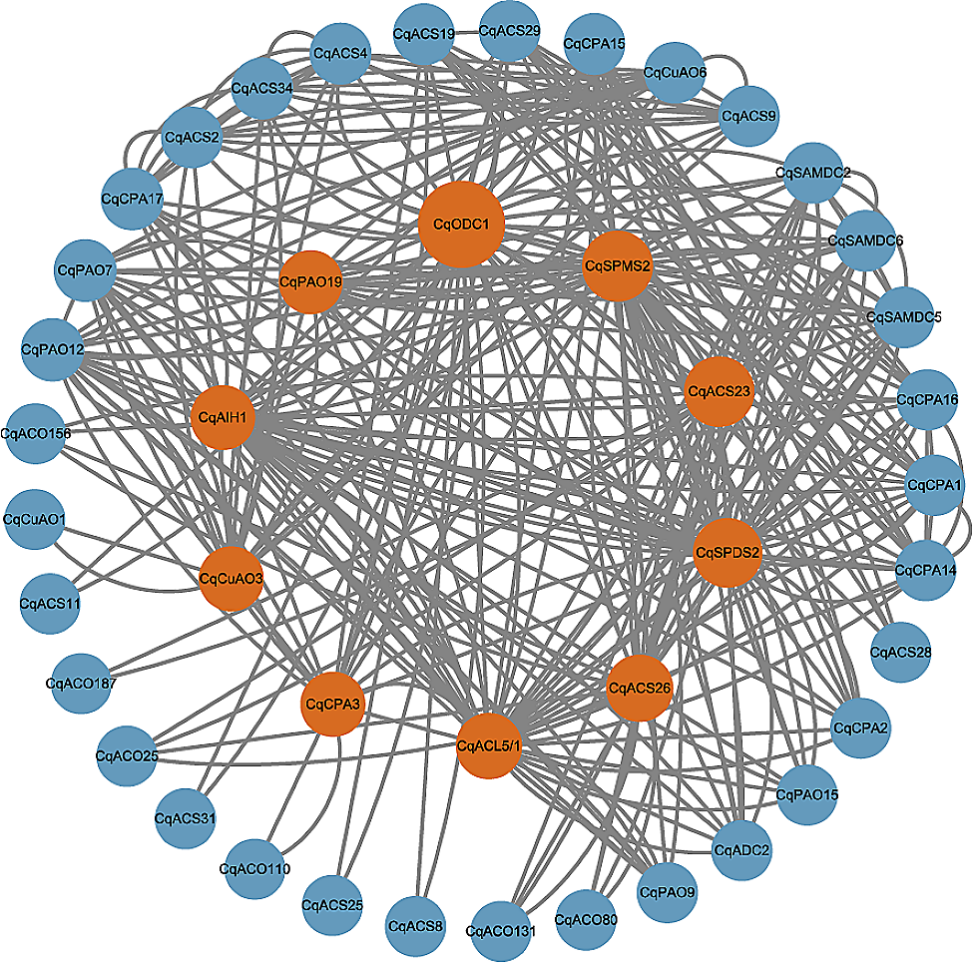
PPI of PA metabolism and ethylene synthesis families

Protein–protein interaction (PPI) network construction of quinoa PA metabolism and ethylene synthesis family proteins were performed using the STRING website (https://string-db.org/) (Fig. 8). The gene had a certain interaction relationship with each other, and 10 genes with high connectivity were screened: *CqODC1*, *CqAIH1*, *CqCPA3*, *CqSPMS2*, *CqSPDS2*, and *CqACL5*/*1* in PA anabolism; *CqCuAO3* and *CqPAO19* in PA catabolism; and *CqACS23* and *CqACS26* in the ethylene synthesis pathway. Among them, *CqODC1* had the highest network connectivity at the center, indicating that this gene is closely related to genes involved in PA metabolism and ethylene synthesis.

### Mantel-test analyses of PA metabolism and ethylene synthesis families

**Fig. 9:**
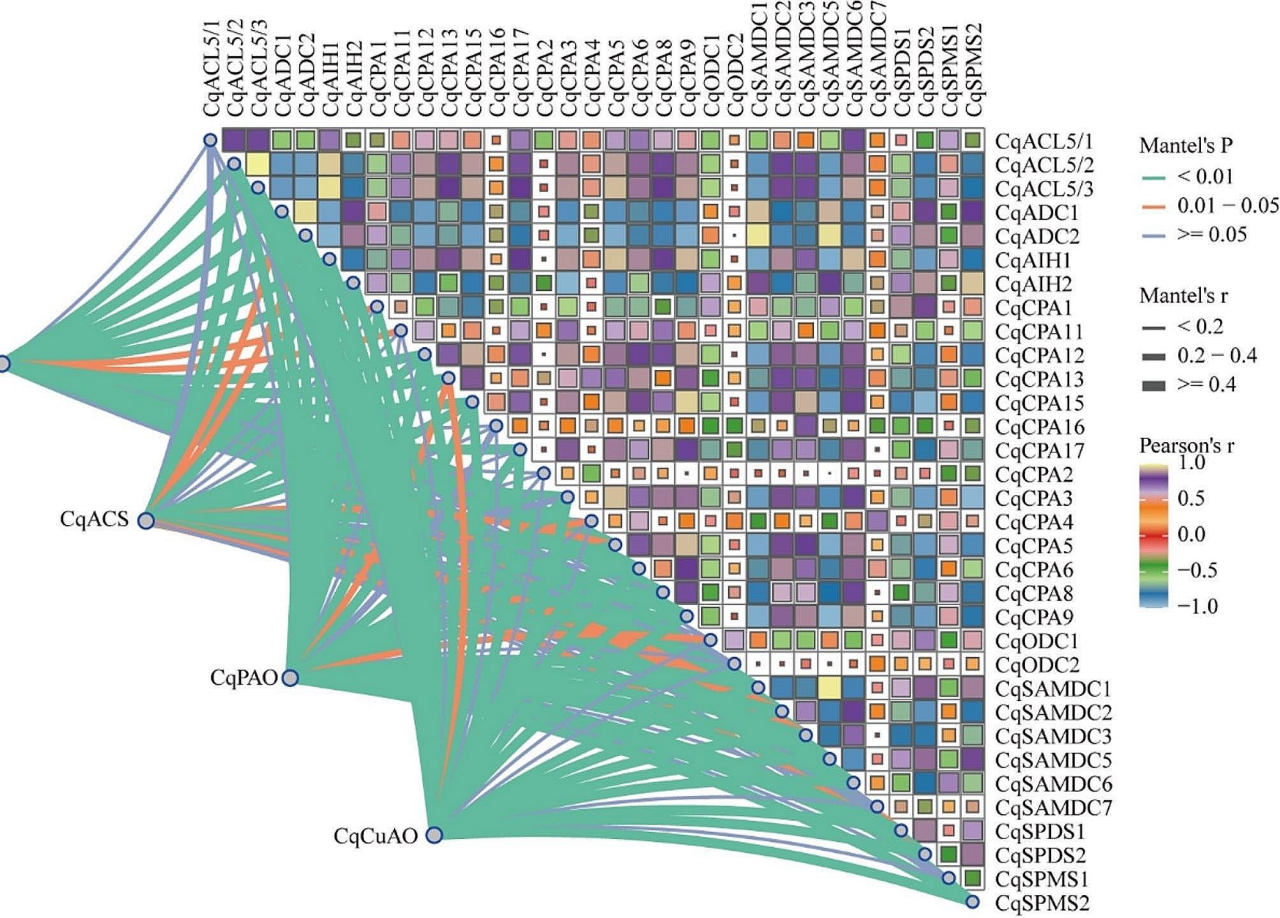
Mantel-test of PA metabolism and ethylene synthesis genes

Mantel test analysis was used to explore further the relationship between PA metabolism and ethylene synthesis gene families (Fig. 9). Different correlations were observed between PA catabolism and ethylene synthesis gene families. The *CqACO*, *CqACS*, *CqCuAO*, and *CqPAO* families showed low significance (*P* < 0.05) for *CqSPMS1*, *CqSAMDC7*, *CqODC2*, *CqCPA2*, *CqCPA16* and C*qACL5/1* of the PA synthesis genes. However, a strong correlation was found between *CqACS* and *CqACL5/1* and between *CqCuAO* and *CqCPA16* (0.2 ≤ *r* < 0.4). The significance of *CqACO* with *CqSPDS1* and *CqCPA4* was low (*P* < 0.05). *CqACS* showed low significance (*P* ≥ 0.05) with *CqSPMS2*, *CqCPA8*, *CqCPA1*, *CqCPA3*, *CqCPA11*, and *CqAIH2*; however, it had a strong correlation with *CqCPA3* (0.2 ≤ *r* < 0.4). *CqCuAO* showed low significance (*P* < 0.05) for *CqODC1*, *CqCPA4*, and *CqCPA11*. In addition, the correlation (*r* ≥ 0.2) and significance (*P* < 0.05) between the genes involved in PA catabolism and ethylene synthesis and the genes involved in PA synthesis were high, indicating a close relationship and mutual influence between these three metabolic pathways.

## Discussion

PAs and ethylene play important roles in plant growth, development, and responses to adverse stress. In this study, we identified the quinoa PA synthesis and catabolic metabolism and ethylene synthesis gene families at the gene level, analyzed the basic family characteristics, and investigated the expression patterns of the gene families of the three metabolic pathways under low-temperature stress.

Members of the same gene family have similar motif and exon-intron structures, and different groups of genes exhibit different structures that have remained conserved through evolution and may play a role in protecting gene integrity. A gene structure with a small number of introns increases the efficiency of gene expression and enables a faster response to external damage, and also implies that the gene is more stable during evolution. In the metabolic pathways of PAs, there were 25 genes that contained less than or equal to 5 introns. Among them, *CqSAMDC3*, *CqSAMDC4*, *CqSAMDC6*, *CqPAO1*, *CqPAO19* ∼ *CqPAO22*, and the *CqADC* gene family did not contain any introns. In the ethylene synthesis pathway, 90% of the genes in the *CqACO* family had less than or equal to 5 introns, which may indicate a faster activation efficiency of these genes. The *SPDS*, *SPMS*, and *ACL5* families are more special, with highly similar protein sequences, so phylogenetic and motif analyses are often employed to determine their homologous sequences. Most plants contain 2 *SPDS* and 1 or 2 SPMS, with the *SPDS* having 9 exons and the *SPMS* having 10 or 11 exons [35]. Quinoa contained two *CqSPDS* and two *CqSPMS* genes, each with 9 and 10 exons respectively, following the gene structure pattern. In addition, the arrangement and size of exons were conserved between the two gene families, indicating that these two classes of enzymes were involved in similar biochemical reactions. The genomic structure of plant *ACL5* genes is also conserved, with independent origin [35]. *ACL5* and SPMS both show substrate specificity for Spermidine and respectively synthesize asymmetric Thermospermine and symmetric Spermine. The Quinoa *CqACL5* family had 3 members, each with 10 exons, consistent with the number and arrangement of exons in other plants of this family, indicating stability during evolution.

A phylogenetic analysis of the PA metabolism and ethylene synthesis genes in quinoa showed that various proteins cluster with homologous proteins in Arabidopsis thaliana, indicating close evolutionary relationships and potentially similar functional roles. The promoter elements of each gene family were diverse, mainly involving stress response, hormone response, developmental response, and light response elements. The more abundant the promoter elements, the faster and broader the coordination in regulating the expression of related genes in time and space, making them more effective in defending against external threats. These genes may play important roles in quinoa growth and development and may be involved in plant defense mechanisms.

Two general pathways are known to synthesize Put in plants: the *ADC* pathway and the *ODC* pathway. *ADC* appears to encode a larger protein than *ODC* [8]. Most plants have the *ODC* pathway, but some Brassicaceae plants such as Arabidopsis thaliana lack this pathway, and the corresponding enzyme activity is defective. Therefore, the *ADC* gene may serve as a compensatory mechanism for the absence of *ODC* in these plants [36]. Changes in gene expression before and after low-temperature stress indicated that the expression trend of *CqODC1* was consistent with *CqADC*, with increased expression levels after temperature stress, suggesting a positive response of these genes to low temperature. Studies have shown that the *ADC* pathway is mainly induced by stress such as low temperature and drought, while the *ODC* pathway is involved in plant growth, organ differentiation, and reproductive stages [37]. For example, overexpression of *AtADC1* in Arabidopsis enhanced cold tolerance, while mutant plants lacking *AtADC* were more sensitive to low temperatures [8]. *ODC* activity significantly increased between the flowering period and fertilization period in tobacco, while using the *ODC* inhibitor Difluoromethylornithine (DFMO) could temporarily halt fruit growth [8].

The expression patterns of *CqAIH2*, *CqCPA1*, *CqSPDS* and *CqSPMS2*, *CqSAMDC1* were consistently upregulated after low-temperature stress, but the opposite was true for other genes in the same family, such as *CqAIH1*, *CqCPA5*, *CqACL17*, and *CqSAMDC6*, probably because when plants are in a low-temperature environment, they will resist external stimuli by increase the PAs content in the body to resist external stimuli, the expression of some genes in the PAs synthesis family, although down-regulated, can be compensated by the high expression and accumulation of other members of the same family, and therefore do not deter the synthesis of PAs [31]. The expression of *CqSAMDC4* was extremely low before and after the low-temperature stress, which may be the result of the expression of the gene was affected by the material varieties, the intensity of the stress, or the tissue site. The expression of CqACL5 family members were all reduced, probably because *CqACL5* competed with *CqSPMS* for the same substrate Spermidine, and quinoa preferentially utilized *CqSPMS* to accumulate Spermine to cope with the external injury during low-temperature stress.

The expression of some genes of the *CqCuAO* and *CqPAO* families was upregulated after low-temperature stress, suggesting that these genes were induced by low temperature. However, *CuAOs* and *PAOs* are mainly responsible for catalyzing PAs and generating a higher amount of H_2_O_2_ [18], so will it reduce the content of PAs, and weaken the plant’s ability to withstand low temperature? In addition reactive oxygen species accumulation is a well-recognized pangenetic marker of abiotic or biotic stress, and it is a matter of existential debate whether H_2_O_2_, formed by PA catabolism, is always pathological or plays a role in cellular signaling [38]. Studies have shown that excess PAs can induce programmed cell death in many different cellular systems [39]. Although the cytotoxicity of polyamines is often attributed to their oxidation, it is well known that high levels of polyamines produce direct toxic effects [39]. Although ROS are deleterious at high concentrations, there is evidence that they are powerful signaling molecules in the adaptive response to stress stimuli [40–42]. In plants, H_2_O_2_ produced by polyamine catabolism is considered to be an important second messenger in the signal transduction network involved in the downstream activation of ion channels and regulation of gene expression, drives peroxidase-mediated oxidative cross-linking of structural cell wall components, contributing to cell wall strengthening during development and under stress conditions, such as wound healing, and low temperature stress [38]. Arabidopsis *AtCuAOα2* and *AtCuAOα3* were involved in xylem differentiation of leaves or roots, and also induced stress-related hormone expression and activated plant defense mechanisms [43]. Overexpression of *Capsicum annuum CaPAO2* and *CaPAO4* in transgenic Arabidopsis significantly increased freezing tolerance, while up-regulating endogenous low temperature responsive genes [44]. Therefore, the increased expression of genes in the *CqCuAO* and *CqPAO* families may not decrease quinoa’s ability to withstand low-temperature damage but may play a positive role in the plant’s response to low-temperature stress.

Ethylene, known as the plant stress hormone, is a gaseous small molecule (C_2_H_4_) that is involved in numerous developmental processes in plants, such as seed germination, cell elongation, tissue differentiation, senescence and abscission of leaves and flowers, and fruit ripening [45, 46]. Moreover, it is also involved in various plant responses to biotic and abiotic stresses [47, 48]. ACS and ACO are the two key enzymes involved in ethylene synthesis. The *CqACO* family has a large number of members, and after low-temperature stress, the family members were categorized into three main groups. The first category was the low-temperature expression type, such as *CqACO165, CqACO106, CqACO118*, and *CqACO190*, whose expression was dramatically upregulated. The second was the low-temperature suppression type, which included *CqACO110, CqACO191*, and *CqACO119*. The third was species-specific types, such as *CqACO9, CqACO25, CqACO131*, and *CqACO132* only appeared in red quinoa, and *CqACO98* was only expressed in white quinoa. The expression of these genes may be related to the variety, but may also be affected by the time of stress or the site of stress. The diversity of *CqACO* gene expression revealed that this family may have highly coordinated and broad functional roles.

The aminopropyl necessary for the synthesis of PAs is catalyzed by *SAMDC* to decompose SAM. SAM is the precursor for PAs and ethylene synthesis. After low temperature stress, the expression level of the *CqSAMDC* gene was upregulated. Theoretically, the PAs content will continue to increase, while the ethylene content is competitively inhibited. In many cases, the PA biosynthesis pathway often competes with ethylene biosynthesis for its common precursor SAM, and PAs interact with ethylene at the level of biosynthesis and physiological effects. However, some studies have failed to observe such competition during flower senescence, cold stress response, or fruit ripening [49]. In this study, PA and ethylene were interlinked and extremely closely related after low-temperature stress, as shown by various analytical methods. However, no obvious antagonistic effect was observed, probably because quinoa synthesized a large amount of PA, the increase in PA also stimulated an increase in the activity of enzymes related to PA catabolic metabolic pathways, which accelerated the decomposition of PAs and produced more reactive oxygen radicals, such as H_2_O_2_. The excessive amount of reactive oxygen radicals accelerated the ACS-catalyzed conversion of ACC to ethylene, increasing ethylene release. In addition, PAs and ethylene compete for the SAM pool during metabolic synthesis, the antagonistic effects of PA and ethylene may only occur when the SAM library capacity is limited, and these two may not show obvious inhibitory effects when the SAM library capacity is sufficient. PA and ethylene are affected by various factors such as stress factors, stress time, tissue site, and developmental period of the plant. The relationship between them cannot be accurately determined from gene family analysis and the expression of PA metabolism genes and ethylene synthesis genes alone. Therefore, we will continue to study the involvement of marker proteins, inhibitors, or the expression of antisense genes to determine the PA and ethylene concentrations to elucidate the relationship between them further.

The structure and expression patterns of members of the same family may be similar, but there are also special members with functional differentiation, for example, the expression of *CqSAMDC1* and *CqSAMDC5* increased significantly after low temperature stress, and correlation analysis showed that they were significantly correlated with PA metabolism and ethylene synthesis pathways, suggesting that they played important roles in the process of resisting cold damage. However, the opposite was true for *CqSAMDC2* and *CqSAMDC3* expression. Therefore making it difficult to evaluate the role of the entire family in plant resistance and growth and development through a single gene [50]. A family is considered as a whole, and by analyzing the differential gene expression and GSEA analysis of the overall trend of gene families in different materials, it is found that after low temperature stress, the expression changes of members of the PAs metabolic and ethylene synthesis gene families were not completely consistent. However, different families of genes showed an overall upregulation trend, indicating that these two metabolisms may participate in the temperature stress process by increasing the expression levels of genes in the metabolic pathways, thus playing a role in resisting stimuli. Members of different families showed different expression levels after low temperature stress, but the overall trend was consistent, which may be the regulation of PAs metabolism and ethylene synthesis genes in quinoa, the molecular basis of both metabolisms to resist low temperature attack.

Different families have different numbers and functions of genes, and there is a complex network of relationships between different genes. Interaction network analysis of the proteins of the PAs metabolism and ethylene synthesis family members showed that *CqODC1*, *CqSPMS2*, and *CqACS23* had a large central connectivity in the proteins, which suggested that they had a great influence on the PAs metabolism and ethylene synthesis family, and thus were involved in the quinoa low-temperature response process. In addition, members of the same family had obvious strong and weak variations in correlation with other families, such as the different correlations between *CqODC1* and *CqODC2* with *CqPAO*, and the inhomogeneity of intergenic expression, which may be related to the complexity, extensiveness, and reinforcement of the function of the PAs family.

## Conclusions

This study conducted a comprehensive genome analysis of the PAs metabolic and ethylene synthesis gene families in quinoa, identifying 37 PAs synthesis genes, 30 PAs catabolism genes, and 227 ethylene synthesis genes, showing a clear family distribution pattern of gene structural characteristics that remained conservative during evolution. By analyzing the transcriptome and proteome data of two quinoa varieties at different temperatures, the expression patterns and protein expression characteristics of the PAs metabolic and ethylene synthesis genes in quinoa were explored. The expression patterns of members of the same family were relatively similar, but differential expression may occur, possibly influenced by factors such as varietal specificity, tissue specificity, and stressor specificity, indicating that this gene family may have multiple stress response mechanisms. Overall, the expression of PAs metabolic genes and ethylene synthesis genes was induced by low temperatures and showed an upregulation trend, playing an important role in the low-temperature response process. The PAs metabolism gene family was closely linked to the ethylene synthesis gene family, and no obvious antagonistic effect was observed. The roles and interactions between the two in plant cold resistance require further exploration and research.

### Electronic supplementary material

Below is the link to the electronic supplementary material.

Supplementary Material 1

## Data Availability

The raw data of transcriptome sequencing can be found in the National Center for Biotechnology Information (NCBI) database with the accession number PRJNA1080447. The proteomic mass spectrometry data were obtained from iProX, accession number IPX0008218001.
