# Supplementary material for: Genome-wide identification of polyamine metabolism and ethylene synthesis genes in Chenopodium quinoa Willd. and their responses to low-temperature stress
Source: BMC Genomics. 2024 Apr 16;25:370. doi: 10.1186/s12864-024-10265-7 (PMC11020822; doi:10.1186/s12864-024-10265-7)
Supplement: Supplementary file 1 — Supplementary Material 1 [file 12864_2024_10265_MOESM1_ESM.docx]

Table S1 Nutrient content of soil

| Nutrient elements | content |
| --- | --- |
| Organic matter content | 47.9 g/kg |
| Effective Phosphorus | 90.73 mg/k |
| Quick-acting Potassium | 101.79 mg/kg |
| Alkaline Nitrogen | 97.6 mg/kg |
| Effective Calcium | 638mg/kg |
| Effective Magnesium | 179 mg/kg |
| Effective Sulfur | 61.42 mg/kg |
| Effective Ferrum | 38 mg/kg |
| Effective Zinc | 4.1 mg/kg |
| Effective Manganese | 5.2 mg/kg |
| Effective Boron | 0.7 mg/kg |
| Effective Cuprum | 1.72 mg/kg |
| Effective Molybdenum | 0.39 mg/kg |

Table S2 Analysis of physicochemical properties of PA metabolic pathway-related gene families

| Gene ID | Protein ID | Protein name | Number of AA | Mw | pI | Ii | Ai | GRAVY | SL |
| --- | --- | --- | --- | --- | --- | --- | --- | --- | --- |
| LOC110705188 | XM_021883043.1 | CqADC1 | 747 | 80138.71 | 5.07 | 38.71 | 87.8 | -0.008 | Cytoplasmic |
| LOC110718863 | XM_021897786.1 | CqADC2 | 746 | 79918.72 | 5.23 | 38.53 | 88.82 | 0.017 | Cytoplasmic |
| LOC110698437 | XM_021875860.1 | CqODC1 | 498 | 54953 | 6.65 | 39.12 | 93.98 | -0.142 | Cytoplasmic |
| LOC110705119 | XM_021882958.1 | CqODC2 | 498 | 54947.08 | 6.87 | 38.73 | 95.14 | -0.136 | Cytoplasmic |
| LOC110698111 | XM_021875517.1 | CqAIH1 | 374 | 41690.96 | 4.99 | 44.4 | 78.21 | -0.396 | Cytoplasmic |
| LOC110726079 | XM_021905533.1 | CqAIH2 | 374 | 41721.97 | 4.99 | 40.49 | 78.72 | -0.429 | Cytoplasmic |
| LOC110683009 | XM_021859344.1 | CqCPA1 | 365 | 40273.84 | 7.68 | 38.65 | 89.59 | -0.234 | Chloroplast |
| LOC110683592 | XM_021859991.1 | CqCPA2 | 348 | 37658.71 | 4.92 | 42.74 | 77.1 | -0.063 | Chloroplast |
| LOC110684236 | XM_021860653.1 | CqCPA3 | 298 | 33246.64 | 5.86 | 36.15 | 79.6 | -0.321 | Cytoplasmic |
| LOC110684371 | XM_021860825.1 | CqCPA4 | 312 | 34854.82 | 6.02 | 32.99 | 74.78 | -0.34 | Cytoplasmic |
| LOC110684630 | XM_021861062.1 | CqCPA5 | 298 | 33228.63 | 5.86 | 37.08 | 80.27 | -0.306 | Cytoplasmic |
| LOC110688104 | XM_021864790.1 | CqCPA6 | 295 | 32944.7 | 5.59 | 25.25 | 81.73 | -0.274 | Cytoplasmic |
| LOC110688104 | XM_021864791.1 | CqCPA7 | 256 | 28673.63 | 5.57 | 29.5 | 75.86 | -0.341 | Cytoplasmic |
| LOC110688144 | XM_021864843.1 | CqCPA8 | 348 | 37639.69 | 4.98 | 46.34 | 77.96 | -0.065 | Chloroplast |
| LOC110689812 | XM_021866625.1 | CqCPA9 | 322 | 35102.14 | 7.07 | 35.85 | 90.62 | 0.011 | Chloroplast |
| LOC110689812 | XM_021866626.1 | CqCPA10 | 304 | 32826.43 | 6.7 | 33.7 | 90.86 | 0.046 | Chloroplast |
| LOC110705390 | XM_021883239.1 | CqCPA11 | 364 | 40037.73 | 8.63 | 41.05 | 90.63 | -0.188 | Chloroplast |
| LOC110710150 | XM_021888408.1 | CqCPA12 | 364 | 40010.58 | 8.42 | 43.36 | 89.84 | -0.213 | Chloroplast |
| LOC110715761 | XM_021894369.1 | CqCPA13 | 365 | 40251.74 | 7.66 | 42.21 | 89.34 | -0.262 | Chloroplast |
| LOC110715761 | XM_021894370.1 | CqCPA14 | 353 | 39159.08 | 9.37 | 45.15 | 85.75 | -0.201 | Nuclear |
| LOC110732890 | XM_021912879.1 | CqCPA15 | 282 | 30825.24 | 6.31 | 37.17 | 91.03 | -0.029 | Chloroplast |
| LOC110736061 | XM_021916210.1 | CqCPA16 | 80 | 9382.99 | 8.09 | 44.32 | 92.75 | -0.105 | Chloroplast |
| LOC110737986 | XM_021918343.1 | CqCPA17 | 415 | 46816.72 | 7.62 | 40.28 | 84.41 | -0.193 | Plasma Membrane |
| LOC110694130 | XM_021871298.1 | CqSPDS1 | 346 | 38115.52 | 4.97 | 47.44 | 87.83 | -0.135 | Cytoplasmic |
| LOC110708466 | XM_021886598.1 | CqSPDS2 | 346 | 38036.42 | 5.01 | 46.69 | 88.38 | -0.116 | Cytoplasmic |
| LOC110696086 | XM_021873365.1 | CqSPMS1 | 365 | 40253.08 | 5.45 | 46.03 | 89.4 | -0.099 | Cytoplasmic |
| LOC110728973 | XM_021908660.1 | CqSPMS2 | 364 | 40330.07 | 5.18 | 48.13 | 89.09 | -0.109 | Cytoplasmic |
| LOC110684982 | XM_021861433.1 | CqACL5/1 | 340 | 37972.86 | 5.25 | 40.43 | 82.85 | -0.293 | Cytoplasmic |
| LOC110705387 | XM_021883236.1 | CqACL5/2 | 337 | 37971.22 | 5.12 | 32.46 | 89.44 | -0.231 | Cytoplasmic |
| LOC110710176 | XM_021888441.1 | CqACL5/3 | 337 | 38073.36 | 5.09 | 33.03 | 90.62 | -0.232 | Cytoplasmic |
| LOC110686006 | XM_021862582.1 | CqSAMDC1 | 376 | 41429.09 | 4.88 | 47.23 | 79.18 | -0.09 | Chloroplast |
| LOC110689723 | XM_021866508.1 | CqSAMDC2 | 401 | 43709.43 | 4.88 | 39.25 | 78.93 | -0.014 | Nuclear |
| LOC110698311 | XM_021875721.1 | CqSAMDC3 | 434 | 48004.75 | 5.46 | 41.67 | 83.92 | 0.038 | Plasma Membrane |
| LOC110715965 | XM_021894584.1 | CqSAMDC4 | 239 | 26039.63 | 5.36 | 46.86 | 89.71 | 0.015 | Plasma Membrane |
| LOC110720713 | XM_021899764.1 | CqSAMDC5 | 377 | 41554.16 | 4.85 | 48 | 79.23 | -0.115 | Chloroplast |
| LOC110721904 | XM_021901137.1 | CqSAMDC6 | 433 | 47816.59 | 5.31 | 40.67 | 84.11 | 0.057 | Plasma Membrane |
| LOC110721905 | XM_021901138.1 | CqSAMDC7 | 400 | 43507.15 | 4.69 | 34.29 | 83.27 | 0.046 | Plasma Membrane |
| LOC110684851 | XM_021861295.1 | CqCuAO1 | 650 | 74422.95 | 9.21 | 38.57 | 80.03 | -0.387 | Plasma Membrane |
| LOC110692771 | XM_021869834.1 | CqCuAO2 | 687 | 77343.23 | 6.34 | 33.91 | 87.21 | -0.291 | Cytoplasmic |
| LOC110696815 | XM_021874150.1 | CqCuAO3 | 281 | 32151.04 | 7.08 | 34.6 | 77.94 | -0.379 | Cytoplasmic |
| LOC110699680 | XM_021877205.1 | CqCuAO4 | 686 | 78193.39 | 6.21 | 38.42 | 83.34 | -0.288 | Plasma Membrane |
| LOC110713448 | XM_021891895.1 | CqCuAO5 | 651 | 74524.09 | 9.14 | 35.74 | 82.6 | -0.374 | Plasma Membrane |
| LOC110732717 | XM_021912706.1 | CqCuAO6 | 721 | 81370.89 | 5.96 | 40.84 | 81.33 | -0.304 | Cytoplasmic |
| LOC110740334 | XM_021857739.1 | CqPAO1 | 852 | 92831.74 | 5.17 | 46.76 | 83.69 | -0.303 | Chloroplast |
| LOC110681743 | XM_021857869.1 | CqPAO2 | 310 | 34557.45 | 5.23 | 34.35 | 89.97 | -0.15 | Cytoplasmic |
| LOC110683809 | XM_021860222.1 | CqPAO3 | 979 | 106650.95 | 7.09 | 41.63 | 84.56 | -0.252 | Chloroplast |
| LOC110686653 | XM_021863257.1 | CqPAO4 | 491 | 55839.54 | 5.79 | 33.02 | 77.09 | -0.375 | Cytoplasmic |
| LOC110686655 | XM_021863258.1 | CqPAO5 | 508 | 58193.19 | 6.56 | 42.45 | 75.87 | -0.45 | Cytoplasmic |
| LOC110686656 | XM_021863259.1 | CqPAO6 | 486 | 55198.99 | 9 | 42.5 | 77.26 | -0.393 | Cytoplasmic |
| LOC110691948 | XM_021868930.1 | CqPAO7 | 489 | 53943.51 | 5.46 | 35.45 | 94.7 | -0.085 | Chloroplast |
| LOC110693215 | XM_021870355.1 | CqPAO8 | 758 | 83602.34 | 6.18 | 36.49 | 94.76 | -0.159 | Cytoplasmic |
| LOC110700862 | XM_021878445.1 | CqPAO9 | 426 | 48215.84 | 9.38 | 39.78 | 85.61 | -0.206 | Plasma Membrane |
| LOC110701998 | XM_021879632.1 | CqPAO10 | 489 | 53893.36 | 5.4 | 36.86 | 93.5 | -0.088 | Chloroplast |
| LOC110702814 | XM_021880561.1 | CqPAO11 | 490 | 55182.74 | 5.23 | 37.64 | 89.76 | -0.26 | Cytoplasmic |
| LOC110702814 | XM_021880566.1 | CqPAO12 | 400 | 45415.57 | 5.11 | 36.01 | 89.45 | -0.268 | Cytoplasmic |
| LOC110705696 | XM_021883607.1 | CqPAO13 | 490 | 54968.33 | 5.17 | 37.21 | 89.73 | -0.258 | Cytoplasmic |
| LOC110708250 | XM_021886336.1 | CqPAO14 | 491 | 54391.12 | 5.31 | 35.52 | 97.13 | -0.044 | Cytoplasmic |
| LOC110708250 | XM_021886337.1 | CqPAO15 | 415 | 46216.78 | 5.1 | 35.53 | 98.46 | -0.068 | Cytoplasmic |
| LOC110709861 | XM_021888127.1 | CqPAO16 | 1939 | 210966.23 | 5.64 | 43.07 | 79.37 | -0.469 | Nuclear |
| LOC110710134 | XM_021888388.1 | CqPAO17 | 982 | 106874.32 | 6.76 | 40.53 | 84.6 | -0.239 | Chloroplast |
| LOC110711120 | XM_021889494.1 | CqPAO18 | 491 | 54277.21 | 5.47 | 30.88 | 98.72 | 0.009 | Cytoplasmic |
| LOC110712904 | XM_021891366.1 | CqPAO19 | 545 | 60318.45 | 5.3 | 39.86 | 83.17 | -0.225 | Cytoplasmic |
| LOC110716826 | XM_021895468.1 | CqPAO20 | 545 | 60516.59 | 5.12 | 39.77 | 81.56 | -0.259 | Cytoplasmic |
| LOC110716841 | XM_021895487.1 | CqPAO21 | 496 | 54634.37 | 8.66 | 35.71 | 97.26 | -0.088 | Plasma Membrane |
| LOC110718643 | XM_021897527.1 | CqPAO22 | 854 | 93190.03 | 5.08 | 45.51 | 83.93 | -0.302 | Chloroplast |
| LOC110723703 | XM_021903052.1 | CqPAO23 | 758 | 83567.25 | 6.52 | 36.1 | 94.89 | -0.175 | Cytoplasmic |
| LOC110724705 | XM_021904155.1 | CqPAO24 | 1938 | 210549.85 | 5.68 | 41.34 | 80.37 | -0.454 | Nuclear |
| LOC110681722 | XM_021857849.1 | CqACS1 | 468 | 52418.95 | 7.99 | 40.05 | 83.78 | -0.251 | Cytoplasmic |
| LOC110683880 | XM_021860289.1 | CqACS2 | 425 | 47081.39 | 5.81 | 38.33 | 95.04 | -0.078 | Cytoplasmic |
| LOC110685603 | XM_021862151.1 | CqACS3 | 468 | 52432.98 | 7.99 | 39.36 | 83.78 | -0.25 | Cytoplasmic |
| LOC110686958 | XM_021863583.1 | CqACS4 | 441 | 48759.35 | 5.3 | 34.52 | 97.73 | 0.012 | Cytoplasmic |
| LOC110688024 | XM_021864688.1 | CqACS5 | 532 | 58424.46 | 5.81 | 47.55 | 86.37 | -0.207 | Chloroplast |
| LOC110689735 | XM_021866518.1 | CqACS6 | 408 | 45514.85 | 5.35 | 50.99 | 84.46 | -0.14 | Cytoplasmic |
| LOC110689738 | XM_021866525.1 | CqACS7 | 404 | 44932.09 | 5.08 | 52.35 | 81.91 | -0.142 | Chloroplast |
| LOC110692488 | XM_021869501.1 | CqACS8 | 545 | 60078.73 | 8.34 | 40.54 | 88.42 | -0.157 | Chloroplast |
| LOC110694240 | XM_021871418.1 | CqACS9 | 476 | 51443.08 | 7.13 | 41.22 | 93.47 | -0.028 | Chloroplast |
| LOC110697172 | XM_021874519.1 | CqACS10 | 553 | 60837.39 | 8.16 | 44.38 | 86.62 | -0.147 | Chloroplast |
| LOC110698724 | XM_021876232.1 | CqACS11 | 394 | 43642.41 | 5.7 | 34.53 | 91.29 | -0.098 | Cytoplasmic |
| LOC110700147 | XM_021877643.1 | CqACS12 | 419 | 46202.13 | 5.67 | 38.41 | 94.92 | -0.032 | Cytoplasmic |
| LOC110700148 | XM_021877644.1 | CqACS13 | 425 | 47039.29 | 6.04 | 42.37 | 92.52 | -0.075 | Cytoplasmic |
| LOC110701458 | XM_021879110.1 | CqACS14 | 418 | 45327.12 | 6.04 | 40.25 | 93.9 | 0.052 | Chloroplast |
| LOC110702562 | XM_021880287.1 | CqACS15 | 547 | 60162.96 | 7.5 | 53.9 | 86.45 | -0.123 | Plasma Membrane |
| LOC110702963 | XM_021880725.1 | CqACS16 | 508 | 55457.5 | 6.81 | 39.93 | 89.49 | -0.091 | Chloroplast |
| LOC110702963 | XM_021880726.1 | CqACS17 | 476 | 51454.98 | 6.81 | 40.93 | 92.44 | -0.055 | Chloroplast |
| LOC110703312 | XM_021881098.1 | CqACS18 | 394 | 43612.28 | 5.56 | 35.14 | 91.04 | -0.105 | Cytoplasmic |
| LOC110709241 | XM_021887459.1 | CqACS19 | 481 | 53184.19 | 6.16 | 38.87 | 86.36 | -0.175 | Chloroplast |
| LOC110712582 | XM_021891050.1 | CqACS20 | 481 | 53219.29 | 6.29 | 38.44 | 85.34 | -0.187 | Cytoplasmic |
| LOC110714040 | XM_021892501.1 | CqACS21 | 435 | 48296.66 | 6.39 | 29.9 | 85.01 | -0.06 | Cytoplasmic |
| LOC110714439 | XM_021892950.1 | CqACS22 | 498 | 56243.14 | 7.25 | 46.92 | 81 | -0.337 | Cytoplasmic |
| LOC110716696 | XM_021895339.1 | CqACS23 | 445 | 50266.87 | 5.27 | 47.04 | 84.18 | -0.291 | Cytoplasmic |
| LOC110716943 | XM_021895593.1 | CqACS24 | 444 | 50117.81 | 5.54 | 49.72 | 84.37 | -0.276 | Plasma Membrane |
| LOC110719398 | XM_021898330.1 | CqACS25 | 548 | 60330.16 | 7.08 | 50.39 | 86.62 | -0.092 | Plasma Membrane |
| LOC110719582 | XM_021898545.1 | CqACS26 | 483 | 53848.55 | 5.94 | 44.19 | 87.81 | -0.128 | Cytoplasmic |
| LOC110722600 | XM_021901864.1 | CqACS27 | 483 | 53939.62 | 5.77 | 44.81 | 87.81 | -0.145 | Cytoplasmic |
| LOC110725343 | XM_021904836.1 | CqACS28 | 448 | 49333.65 | 6.39 | 31.23 | 83.46 | -0.06 | Chloroplast |
| LOC110726705 | XM_021906177.1 | CqACS29 | 512 | 56527.84 | 6.36 | 47.79 | 90.86 | -0.135 | Chloroplast |
| LOC110726706 | XM_021906179.1 | CqACS30 | 532 | 58405.39 | 5.68 | 46.09 | 86 | -0.209 | Chloroplast |
| LOC110727989 | XM_021907589.1 | CqACS31 | 362 | 40096.71 | 5.32 | 49.38 | 88.15 | -0.095 | Chloroplast |
| LOC110727990 | XM_021907590.1 | CqACS32 | 406 | 45170.41 | 5.02 | 51.75 | 83.2 | -0.116 | Cytoplasmic |
| LOC110731584 | XM_021911452.1 | CqACS33 | 497 | 55985.93 | 7.23 | 45.54 | 83.52 | -0.288 | Cytoplasmic |
| LOC110732609 | XM_021912571.1 | CqACS34 | 447 | 48856.17 | 5.9 | 27.45 | 94.23 | -0.022 | Chloroplast |
| LOC110736023 | XM_021916182.1 | CqACS35 | 407 | 44126.97 | 7.55 | 40.11 | 94.74 | 0.038 | Chloroplast |
| LOC110681963 | XM_021858152.1 | CqACO1 | 347 | 38626.64 | 5.24 | 47.97 | 85.65 | -0.369 | Nuclear |
| LOC110682098 | XM_021858337.1 | CqACO2 | 353 | 39016.73 | 6.35 | 38.36 | 83.4 | -0.233 | Cytoplasmic |
| LOC110682099 | XM_021858338.1 | CqACO3 | 319 | 35515.51 | 6.21 | 30.69 | 91.32 | -0.264 | Cytoplasmic |
| LOC110682099 | XM_021858339.1 | CqACO4 | 315 | 35028.04 | 6.37 | 31.57 | 92.48 | -0.228 | Cytoplasmic |
| LOC110682339 | XM_021858669.1 | CqACO5 | 330 | 36725.11 | 5.72 | 20.15 | 91.24 | -0.208 | Cytoplasmic |
| LOC110682339 | XM_021858670.1 | CqACO6 | 329 | 36656.99 | 6.56 | 22.08 | 90.33 | -0.267 | Cytoplasmic |
| LOC110682587 | XM_021858942.1 | CqACO7 | 368 | 40946.97 | 5.79 | 27.1 | 86.44 | -0.194 | Cytoplasmic |
| LOC110683123 | XM_021859474.1 | CqACO8 | 328 | 37644.99 | 5.83 | 43.21 | 89.39 | -0.432 | Nuclear |
| LOC110684949 | XM_021861394.1 | CqACO9 | 372 | 41927.26 | 6.77 | 39.11 | 84.03 | -0.313 | Cytoplasmic |
| LOC110684996 | XM_021861450.1 | CqACO10 | 372 | 42011.07 | 6.42 | 44.33 | 85.65 | -0.366 | Cytoplasmic |
| LOC110685386 | XM_021861915.1 | CqACO11 | 395 | 44904.89 | 5.82 | 33.05 | 71.29 | -0.447 | Nuclear |
| LOC110685386 | XM_021861917.1 | CqACO12 | 389 | 44176.14 | 5.92 | 31.53 | 71.39 | -0.443 | Nuclear |
| LOC110685944 | XM_021862500.1 | CqACO13 | 320 | 36386.24 | 4.94 | 42.98 | 81.28 | -0.487 | Cytoplasmic |
| LOC110686439 | XM_021863061.1 | CqACO14 | 372 | 42127.25 | 5.53 | 38.88 | 87.77 | -0.407 | Cytoplasmic |
| LOC110686440 | XM_021863062.1 | CqACO15 | 365 | 41258 | 5.7 | 40.2 | 83.64 | -0.456 | Cytoplasmic |
| LOC110687417 | XM_021864065.1 | CqACO16 | 319 | 36183.45 | 5.29 | 31.98 | 80.69 | -0.458 | Cytoplasmic |
| LOC110688242 | XM_021864964.1 | CqACO17 | 349 | 40148.89 | 6.7 | 35.6 | 92.09 | -0.379 | Cytoplasmic |
| LOC110689232 | XM_021865962.1 | CqACO18 | 360 | 40829.94 | 6.02 | 43.73 | 88.78 | -0.276 | Cytoplasmic |
| LOC110689448 | XM_021866241.1 | CqACO19 | 276 | 31168.7 | 5.05 | 31.87 | 90.07 | -0.303 | Cytoplasmic |
| LOC110689873 | XM_021866708.1 | CqACO20 | 371 | 42113.21 | 5.43 | 38.62 | 91.37 | -0.286 | Cytoplasmic |
| LOC110689942 | XM_021866764.1 | CqACO21 | 363 | 41283.48 | 5.58 | 35.93 | 90.77 | -0.301 | Cytoplasmic |
| LOC110689950 | XM_021866771.1 | CqACO22 | 355 | 40430.29 | 5.26 | 41.61 | 89.55 | -0.382 | Cytoplasmic |
| LOC110690949 | XM_021867868.1 | CqACO23 | 348 | 39375.72 | 5.34 | 31.14 | 78.42 | -0.411 | Cytoplasmic |
| LOC110690949 | XM_021867869.1 | CqACO24 | 346 | 39205.51 | 5.34 | 31.51 | 77.75 | -0.431 | Cytoplasmic |
| LOC110691904 | XM_021868881.1 | CqACO25 | 282 | 32156.73 | 5.35 | 32.58 | 87.8 | -0.434 | Cytoplasmic |
| LOC110692918 | XM_021869995.1 | CqACO26 | 341 | 39183.5 | 5.55 | 46.15 | 81.73 | -0.487 | Cytoplasmic |
| LOC110692918 | XM_021869996.1 | CqACO27 | 339 | 38908.19 | 5.55 | 46.59 | 82.21 | -0.488 | Cytoplasmic |
| LOC110693057 | XM_021870182.1 | CqACO28 | 366 | 41653.49 | 5.36 | 38.44 | 88.42 | -0.429 | Cytoplasmic |
| LOC110693060 | XM_021870185.1 | CqACO29 | 373 | 42294.26 | 5.22 | 35.53 | 92.49 | -0.282 | Cytoplasmic |
| LOC110693061 | XM_021870186.1 | CqACO30 | 391 | 44601.84 | 6.12 | 41.14 | 90.69 | -0.343 | Nuclear |
| LOC110693062 | XM_021870187.1 | CqACO31 | 377 | 42659.23 | 5.73 | 36.81 | 86.6 | -0.363 | Cytoplasmic |
| LOC110693062 | XM_021870188.1 | CqACO32 | 376 | 42739.49 | 6.14 | 40.12 | 86.84 | -0.38 | Cytoplasmic |
| LOC110693063 | XM_021870189.1 | CqACO33 | 370 | 41885.71 | 5.61 | 41.18 | 92.65 | -0.282 | Cytoplasmic |
| LOC110693063 | XM_021870190.1 | CqACO34 | 370 | 41984.11 | 5.58 | 38.96 | 95.32 | -0.23 | Cytoplasmic |
| LOC110693063 | XM_021870192.1 | CqACO35 | 369 | 41719.47 | 5.47 | 40.63 | 94.23 | -0.27 | Cytoplasmic |
| LOC110693494 | XM_021870606.1 | CqACO36 | 339 | 38423.03 | 6.28 | 42.27 | 85.4 | -0.492 | Cytoplasmic |
| LOC110694697 | XM_021871864.1 | CqACO37 | 370 | 41597.51 | 5.34 | 40.99 | 84.62 | -0.431 | Cytoplasmic |
| LOC110694757 | XM_021871931.1 | CqACO38 | 297 | 32943.94 | 5.72 | 52.54 | 98.82 | -0.122 | Cytoplasmic |
| LOC110695019 | XM_021872212.1 | CqACO39 | 380 | 42846.27 | 5.81 | 33.92 | 94.42 | -0.169 | Cytoplasmic |
| LOC110695126 | XM_021872346.1 | CqACO40 | 346 | 38485.52 | 5.58 | 47.16 | 86.18 | -0.341 | Nuclear |
| LOC110695366 | XM_021872610.1 | CqACO41 | 353 | 39061.72 | 5.84 | 41.53 | 85.07 | -0.203 | Cytoplasmic |
| LOC110695786 | XM_021873019.1 | CqACO42 | 387 | 43305.67 | 6.4 | 37.69 | 88.55 | -0.291 | Cytoplasmic |
| LOC110695790 | XM_021873021.1 | CqACO43 | 368 | 41654.35 | 5.22 | 40.88 | 89.48 | -0.311 | Cytoplasmic |
| LOC110695795 | XM_021873027.1 | CqACO44 | 362 | 40730.27 | 5.5 | 32.54 | 89.09 | -0.32 | Cytoplasmic |
| LOC110697144 | XM_021874490.1 | CqACO45 | 387 | 44070.96 | 6.37 | 39.41 | 71.24 | -0.5 | Nuclear |
| LOC110697292 | XM_021874665.1 | CqACO46 | 393 | 44232.44 | 6.32 | 34.75 | 82.82 | -0.358 | Cytoplasmic |
| LOC110697430 | XM_021874807.1 | CqACO47 | 366 | 41354.96 | 5.57 | 30.08 | 82.51 | -0.386 | Cytoplasmic |
| LOC110698561 | XM_021876006.1 | CqACO48 | 354 | 40649.11 | 5.73 | 40.11 | 84.52 | -0.506 | Cytoplasmic |
| LOC110698563 | XM_021876009.1 | CqACO49 | 343 | 39460.89 | 5.37 | 36.25 | 83.24 | -0.474 | Cytoplasmic |
| LOC110698722 | XM_021876230.1 | CqACO50 | 386 | 42715.87 | 8.26 | 31.65 | 93.99 | -0.214 | Cytoplasmic |
| LOC110699285 | XM_021876787.1 | CqACO51 | 358 | 40931.71 | 6.52 | 41.42 | 85.39 | -0.458 | Cytoplasmic |
| LOC110700794 | XM_021878388.1 | CqACO52 | 347 | 39094.55 | 5.08 | 39.4 | 89.57 | -0.222 | Cytoplasmic |
| LOC110701517 | XM_021879154.1 | CqACO53 | 382 | 44191.22 | 6.64 | 39.72 | 69.42 | -0.49 | Cytoplasmic |
| LOC110701517 | XM_021879155.1 | CqACO54 | 379 | 43765.69 | 6.32 | 37.48 | 68.94 | -0.482 | Cytoplasmic |
| LOC110701693 | XM_021879322.1 | CqACO55 | 327 | 36087.15 | 5.37 | 24.24 | 89.17 | -0.235 | Cytoplasmic |
| LOC110701704 | XM_021879330.1 | CqACO56 | 336 | 37339.05 | 8.72 | 17.51 | 85.36 | -0.29 | Cytoplasmic |
| LOC110701716 | XM_021879343.1 | CqACO57 | 319 | 35467.65 | 5.54 | 28.26 | 89.22 | -0.214 | Cytoplasmic |
| LOC110701716 | XM_021879344.1 | CqACO58 | 277 | 31099.65 | 5.92 | 25.98 | 88.3 | -0.271 | Cytoplasmic |
| LOC110701957 | XM_021879578.1 | CqACO59 | 322 | 36589.89 | 5.39 | 30.38 | 89.32 | -0.398 | Cytoplasmic |
| LOC110702537 | XM_021880258.1 | CqACO60 | 369 | 41790.5 | 5.27 | 42.4 | 87.18 | -0.5 | Cytoplasmic |
| LOC110702736 | XM_021880483.1 | CqACO61 | 372 | 42305.39 | 5.45 | 32.82 | 94.09 | -0.269 | Cytoplasmic |
| LOC110702737 | XM_021880484.1 | CqACO62 | 379 | 43194.39 | 6.08 | 39.74 | 90.03 | -0.328 | Cytoplasmic |
| LOC110703310 | XM_021881096.1 | CqACO63 | 369 | 41034.89 | 8.71 | 34 | 90.11 | -0.27 | Nuclear |
| LOC110703926 | XM_021881721.1 | CqACO64 | 350 | 39381.77 | 5.8 | 21.58 | 84.97 | -0.407 | Cytoplasmic |
| LOC110704346 | XM_021882130.1 | CqACO65 | 364 | 41643.62 | 5.42 | 42.65 | 82.97 | -0.356 | Cytoplasmic |
| LOC110704435 | XM_021882217.1 | CqACO66 | 376 | 42383.2 | 5.52 | 42.66 | 89.18 | -0.376 | Cytoplasmic |
| LOC110704447 | XM_021882230.1 | CqACO67 | 315 | 36360.52 | 5.9 | 48.86 | 81.68 | -0.402 | Cytoplasmic |
| LOC110704990 | XM_021882801.1 | CqACO68 | 356 | 40848.27 | 5.33 | 38.75 | 84.58 | -0.488 | Cytoplasmic |
| LOC110707600 | XM_021885628.1 | CqACO69 | 354 | 40300.07 | 6.27 | 40.73 | 80.96 | -0.318 | Cytoplasmic |
| LOC110707701 | XM_021885736.1 | CqACO70 | 348 | 39390.7 | 5.18 | 34.9 | 81.78 | -0.39 | Cytoplasmic |
| LOC110707701 | XM_021885737.1 | CqACO71 | 346 | 39220.49 | 5.18 | 35.29 | 81.13 | -0.41 | Cytoplasmic |
| LOC110708028 | XM_021886130.1 | CqACO72 | 360 | 41521.43 | 5.97 | 44.71 | 88.53 | -0.483 | Nuclear |
| LOC110708033 | XM_021886132.1 | CqACO73 | 351 | 40054.04 | 5.57 | 35.68 | 86.15 | -0.253 | Cytoplasmic |
| LOC110708034 | XM_021886133.1 | CqACO74 | 351 | 39838.51 | 5.28 | 41.79 | 84.47 | -0.337 | Cytoplasmic |
| LOC110708783 | XM_021887004.1 | CqACO75 | 399 | 44738.07 | 6.02 | 38.39 | 89.62 | -0.257 | Cytoplasmic |
| LOC110708783 | XM_021887006.1 | CqACO76 | 357 | 40192.74 | 5.64 | 37.83 | 90.87 | -0.329 | Cytoplasmic |
| LOC110711051 | XM_021889427.1 | CqACO77 | 358 | 40901.69 | 6.52 | 40.88 | 85.39 | -0.456 | Cytoplasmic |
| LOC110712216 | XM_021890677.1 | CqACO78 | 356 | 39960 | 5.73 | 32.32 | 90.56 | -0.181 | Cytoplasmic |
| LOC110712191 | XM_021890681.1 | CqACO79 | 340 | 38021.83 | 8.77 | 39.87 | 89.15 | -0.198 | Nuclear |
| LOC110712995 | XM_021891454.1 | CqACO80 | 321 | 36832.04 | 7.63 | 38.28 | 75.92 | -0.465 | Cytoplasmic |
| LOC110713641 | XM_021892131.1 | CqACO81 | 366 | 41177.72 | 5.75 | 31.64 | 83.06 | -0.378 | Cytoplasmic |
| LOC110714071 | XM_021892536.1 | CqACO82 | 319 | 36224.32 | 5.59 | 47.58 | 74.48 | -0.329 | Cytoplasmic |
| LOC110714529 | XM_021893063.1 | CqACO83 | 346 | 39409.91 | 6.04 | 46.5 | 84.48 | -0.549 | Cytoplasmic |
| LOC110714551 | XM_021893088.1 | CqACO84 | 389 | 44201.3 | 6.32 | 29.13 | 71.65 | -0.441 | Cytoplasmic |
| LOC110714562 | XM_021893101.1 | CqACO85 | 395 | 43896.26 | 6.36 | 29.9 | 89.22 | -0.275 | Cytoplasmic |
| LOC110714563 | XM_021893102.1 | CqACO86 | 368 | 41491.23 | 5.53 | 41.56 | 91.87 | -0.299 | Cytoplasmic |
| LOC110714563 | XM_021893103.1 | CqACO87 | 366 | 41487.12 | 6.28 | 45.39 | 89.73 | -0.398 | Nuclear |
| LOC110714564 | XM_021893104.1 | CqACO88 | 365 | 41208.87 | 5.14 | 38.87 | 90.22 | -0.297 | Cytoplasmic |
| LOC110714565 | XM_021893105.1 | CqACO89 | 362 | 40681.21 | 5.53 | 34.06 | 88.04 | -0.329 | Cytoplasmic |
| LOC110715086 | XM_021893666.1 | CqACO90 | 361 | 40728.54 | 5.49 | 42.32 | 89.34 | -0.29 | Cytoplasmic |
| LOC110715111 | XM_021893688.1 | CqACO91 | 372 | 41996.25 | 6.48 | 28.16 | 92.74 | -0.296 | Cytoplasmic |
| LOC110715271 | XM_021893835.1 | CqACO92 | 384 | 43461.06 | 5.91 | 41.15 | 92.89 | -0.287 | Cytoplasmic |
| LOC110715411 | XM_021893993.1 | CqACO93 | 385 | 43791.05 | 6.47 | 44.49 | 80.03 | -0.35 | Nuclear |
| LOC110715491 | XM_021894069.1 | CqACO94 | 312 | 35791.91 | 5.42 | 37.72 | 79.29 | -0.32 | Cytoplasmic |
| LOC110715662 | XM_021894256.1 | CqACO95 | 343 | 39008.59 | 5.09 | 47.02 | 86.65 | -0.401 | Nuclear |
| LOC110716847 | XM_021895493.1 | CqACO96 | 302 | 34625.35 | 6.42 | 33.77 | 71.36 | -0.615 | Cytoplasmic |
| LOC110716896 | XM_021895549.1 | CqACO97 | 374 | 42334.78 | 6.12 | 42.06 | 95.88 | -0.298 | Cytoplasmic |
| LOC110717552 | XM_021896283.1 | CqACO98 | 371 | 42168 | 5.07 | 32.96 | 91.91 | -0.255 | Cytoplasmic |
| LOC110717628 | XM_021896375.1 | CqACO99 | 371 | 41869.48 | 5.34 | 38.15 | 88.73 | -0.299 | Cytoplasmic |
| LOC110717756 | XM_021896520.1 | CqACO100 | 372 | 42634.73 | 5.86 | 39.71 | 96.37 | -0.335 | Cytoplasmic |
| LOC110717938 | XM_021896737.1 | CqACO101 | 366 | 41549.43 | 5.3 | 38.83 | 88.14 | -0.414 | Cytoplasmic |
| LOC110718176 | XM_021896983.1 | CqACO102 | 369 | 41871.61 | 5.67 | 34.6 | 87.07 | -0.42 | Cytoplasmic |
| LOC110719267 | XM_021898160.1 | CqACO103 | 359 | 40480.16 | 5.37 | 42.29 | 89.64 | -0.382 | Cytoplasmic |
| LOC110722025 | XM_021901248.1 | CqACO104 | 313 | 36171.67 | 8.16 | 44.62 | 92.17 | -0.334 | Cytoplasmic |
| LOC110722832 | XM_021902132.1 | CqACO105 | 315 | 36003.84 | 5.29 | 47.44 | 79.87 | -0.405 | Cytoplasmic |
| LOC110722833 | XM_021902133.1 | CqACO106 | 312 | 35578.49 | 5.42 | 41.16 | 86.89 | -0.356 | Cytoplasmic |
| LOC110722834 | XM_021902134.1 | CqACO107 | 312 | 35733.67 | 4.99 | 42.71 | 86.28 | -0.303 | Cytoplasmic |
| LOC110723117 | XM_021902417.1 | CqACO108 | 356 | 39988.81 | 5.77 | 34.48 | 87.56 | -0.233 | Cytoplasmic |
| LOC110723209 | XM_021902550.1 | CqACO109 | 234 | 27104.85 | 5.35 | 53.03 | 81.2 | -0.495 | Cytoplasmic |
| LOC110724214 | XM_021903626.1 | CqACO110 | 301 | 33416.27 | 5.82 | 41.09 | 90.96 | -0.195 | Cytoplasmic |
| LOC110724224 | XM_021903635.1 | CqACO111 | 294 | 32680.46 | 5.12 | 45.7 | 97.18 | -0.132 | Cytoplasmic |
| LOC110724233 | XM_021903645.1 | CqACO112 | 295 | 32893.84 | 6.09 | 48.88 | 98.14 | -0.143 | Cytoplasmic |
| LOC110724781 | XM_021904247.1 | CqACO113 | 370 | 41620.5 | 5.34 | 41.56 | 83.57 | -0.444 | Cytoplasmic |
| LOC110724886 | XM_021904384.1 | CqACO114 | 454 | 51969.92 | 8.32 | 43.54 | 90.33 | -0.28 | Cytoplasmic |
| LOC110724886 | XM_021904385.1 | CqACO115 | 353 | 40012.94 | 5.31 | 35.5 | 96.57 | -0.235 | Cytoplasmic |
| LOC110725014 | XM_021904494.1 | CqACO116 | 380 | 43063.49 | 6.04 | 33.75 | 96.24 | -0.192 | Cytoplasmic |
| LOC110725016 | XM_021904495.1 | CqACO117 | 323 | 36159.72 | 4.82 | 36.78 | 107.49 | 0.05 | Plasma Membrane |
| LOC110725246 | XM_021904734.1 | CqACO118 | 356 | 40245.26 | 5.21 | 33.4 | 87.05 | -0.24 | Cytoplasmic |
| LOC110725334 | XM_021904825.1 | CqACO119 | 346 | 39496.05 | 6.04 | 44.05 | 85.61 | -0.544 | Cytoplasmic |
| LOC110725374 | XM_021904861.1 | CqACO120 | 333 | 38051.19 | 5.84 | 41.73 | 86.61 | -0.468 | Nuclear |
| LOC110725468 | XM_021904941.1 | CqACO121 | 383 | 43525.28 | 5.64 | 29.72 | 76.37 | -0.448 | Cytoplasmic |
| LOC110725534 | XM_021904996.1 | CqACO122 | 403 | 46896.82 | 6.28 | 40.23 | 91.86 | -0.334 | Cytoplasmic |
| LOC110725535 | XM_021904997.1 | CqACO123 | 397 | 45167.48 | 5.75 | 44.19 | 103.05 | -0.122 | Cytoplasmic |
| LOC110725535 | XM_021904998.1 | CqACO124 | 352 | 40242.47 | 5.79 | 38.25 | 99.09 | -0.18 | Cytoplasmic |
| LOC110725536 | XM_021904999.1 | CqACO125 | 367 | 41773.81 | 5.47 | 40.54 | 84.82 | -0.269 | Cytoplasmic |
| LOC110725539 | XM_021905000.1 | CqACO126 | 353 | 41113.76 | 5.29 | 37.93 | 78.39 | -0.527 | Cytoplasmic |
| LOC110725540 | XM_021905001.1 | CqACO127 | 351 | 39857.58 | 5.36 | 42.06 | 83.08 | -0.348 | Cytoplasmic |
| LOC110725541 | XM_021905002.1 | CqACO128 | 349 | 39749.65 | 5.5 | 35.82 | 85.3 | -0.343 | Cytoplasmic |
| LOC110725554 | XM_021905007.1 | CqACO129 | 358 | 41120.97 | 5.53 | 48.12 | 89.25 | -0.432 | Cytoplasmic |
| LOC110725555 | XM_021905008.1 | CqACO130 | 357 | 41257.01 | 5.65 | 45.98 | 87.62 | -0.52 | Nuclear |
| LOC110725726 | XM_021905194.1 | CqACO131 | 321 | 36416.29 | 4.91 | 44.96 | 79.22 | -0.488 | Cytoplasmic |
| LOC110726535 | XM_021906017.1 | CqACO132 | 388 | 43729.15 | 5.49 | 43.97 | 90.93 | -0.184 | Cytoplasmic |
| LOC110727607 | XM_021907191.1 | CqACO133 | 389 | 44855.28 | 5.86 | 50.55 | 83.19 | -0.33 | Extracellular |
| LOC110728057 | XM_021907672.1 | CqACO134 | 380 | 42881.96 | 5.57 | 36.54 | 84.42 | -0.309 | Cytoplasmic |
| LOC110728820 | XM_021908488.1 | CqACO135 | 363 | 41246.5 | 5.66 | 37.04 | 97.77 | -0.24 | Cytoplasmic |
| LOC110728825 | XM_021908492.1 | CqACO136 | 371 | 42164.26 | 5.8 | 39.26 | 89.51 | -0.327 | Cytoplasmic |
| LOC110728826 | XM_021908493.1 | CqACO137 | 367 | 41403.61 | 5.61 | 37.53 | 92.7 | -0.332 | Cytoplasmic |
| LOC110728827 | XM_021908494.1 | CqACO138 | 360 | 41045.24 | 5.42 | 44.87 | 93.42 | -0.347 | Cytoplasmic |
| LOC110729743 | XM_021909528.1 | CqACO139 | 387 | 44081.77 | 5.96 | 37.11 | 69.97 | -0.521 | Nuclear |
| LOC110729756 | XM_021909542.1 | CqACO140 | 343 | 39473.18 | 5.59 | 45.77 | 84.08 | -0.525 | Nuclear |
| LOC110729860 | XM_021909647.1 | CqACO141 | 380 | 43710.17 | 6.68 | 52.17 | 81.55 | -0.35 | Nuclear |
| LOC110729859 | XM_021909650.1 | CqACO142 | 358 | 40838.7 | 5.4 | 39.65 | 87.15 | -0.365 | Cytoplasmic |
| LOC110729868 | XM_021909659.1 | CqACO143 | 349 | 40214.93 | 5.38 | 41.55 | 88.25 | -0.491 | Cytoplasmic |
| LOC110729876 | XM_021909663.1 | CqACO144 | 311 | 35538.53 | 5.41 | 40.23 | 79.23 | -0.339 | Cytoplasmic |
| LOC110729880 | XM_021909669.1 | CqACO145 | 344 | 39499.26 | 5.83 | 45.95 | 83.55 | -0.525 | Nuclear |
| LOC110729890 | XM_021909679.1 | CqACO146 | 343 | 38963.55 | 5.15 | 45.04 | 86.65 | -0.402 | Nuclear |
| LOC110729983 | XM_021909770.1 | CqACO147 | 341 | 39427.44 | 5.17 | 40.43 | 95.69 | -0.211 | Cytoplasmic |
| LOC110730141 | XM_021909915.1 | CqACO148 | 355 | 40543.28 | 5.72 | 42.29 | 85.89 | -0.358 | Cytoplasmic |
| LOC110730548 | XM_021910357.1 | CqACO149 | 363 | 41184.29 | 6.12 | 31.78 | 92.12 | -0.324 | Cytoplasmic |
| LOC110730727 | XM_021910549.1 | CqACO150 | 352 | 39990.67 | 5.54 | 30.37 | 86.7 | -0.272 | Cytoplasmic |
| LOC110730729 | XM_021910551.1 | CqACO151 | 354 | 40120.09 | 6.67 | 30.92 | 87.01 | -0.232 | Cytoplasmic |
| LOC110730947 | XM_021910794.1 | CqACO152 | 349 | 40325.07 | 5.56 | 39.24 | 86.02 | -0.539 | Cytoplasmic |
| LOC110730960 | XM_021910804.1 | CqACO153 | 350 | 40270.91 | 5.89 | 44.87 | 82.71 | -0.563 | Nuclear |
| LOC110731036 | XM_021910888.1 | CqACO154 | 353 | 40407.53 | 5.96 | 34.56 | 90.59 | -0.288 | Cytoplasmic |
| LOC110731044 | XM_021910896.1 | CqACO155 | 358 | 41045.99 | 6.18 | 47.4 | 86.56 | -0.51 | Nuclear |
| LOC110731284 | XM_021911126.1 | CqACO156 | 318 | 36108.47 | 5.46 | 32.71 | 82.17 | -0.435 | Cytoplasmic |
| LOC110731393 | XM_021911255.1 | CqACO157 | 374 | 42629.69 | 7.95 | 42.04 | 79.47 | -0.393 | Nuclear |
| LOC110731393 | XM_021911256.1 | CqACO158 | 354 | 40408.04 | 6.51 | 42.13 | 79.55 | -0.382 | Nuclear |
| LOC110731394 | XM_021911257.1 | CqACO159 | 353 | 40159.82 | 7.12 | 43.99 | 84.73 | -0.34 | Plasma Membrane |
| LOC110731530 | XM_021911381.1 | CqACO160 | 319 | 36182.36 | 5.73 | 47.12 | 74.2 | -0.307 | Cytoplasmic |
| LOC110732253 | XM_021912167.1 | CqACO161 | 251 | 28528.42 | 4.87 | 49.66 | 92.07 | -0.288 | Cytoplasmic |
| LOC110732367 | XM_021912299.1 | CqACO162 | 350 | 39641.2 | 5.6 | 43.39 | 87.49 | -0.265 | Extracellular |
| LOC110732367 | XM_021912300.1 | CqACO163 | 316 | 35851.71 | 5.06 | 42.83 | 87.97 | -0.301 | Cytoplasmic |
| LOC110732370 | XM_021912303.1 | CqACO164 | 312 | 35707.74 | 5.27 | 40.23 | 83.78 | -0.327 | Cytoplasmic |
| LOC110732909 | XM_021912896.1 | CqACO165 | 370 | 41982 | 5.98 | 46.15 | 89.03 | -0.311 | Cytoplasmic |
| LOC110732934 | XM_021912919.1 | CqACO166 | 884 | 100879.54 | 5.77 | 38.56 | 84.5 | -0.348 | Cytoplasmic |
| LOC110732935 | XM_021912920.1 | CqACO167 | 337 | 38209.84 | 5.95 | 44.18 | 90.24 | -0.259 | Cytoplasmic |
| LOC110732936 | XM_021912921.1 | CqACO168 | 337 | 38058.45 | 5.21 | 36.99 | 87.36 | -0.218 | Cytoplasmic |
| LOC110732989 | XM_021912986.1 | CqACO169 | 326 | 36034.2 | 7.74 | 47.6 | 89.42 | -0.201 | Mitochondrial |
| LOC110733133 | XM_021913156.1 | CqACO170 | 341 | 39174.07 | 6.27 | 35.75 | 82.58 | -0.332 | Cytoplasmic |
| LOC110733142 | XM_021913166.1 | CqACO171 | 364 | 41587.43 | 5.94 | 52.63 | 84.7 | -0.456 | Nuclear |
| LOC110733449 | XM_021913502.1 | CqACO172 | 358 | 40893.54 | 5.68 | 40.41 | 85.39 | -0.476 | Cytoplasmic |
| LOC110733452 | XM_021913509.1 | CqACO173 | 365 | 41222.01 | 5.71 | 38.19 | 84.16 | -0.443 | Cytoplasmic |
| LOC110733463 | XM_021913519.1 | CqACO174 | 365 | 41193.96 | 5.62 | 38.19 | 84.16 | -0.44 | Cytoplasmic |
| LOC110733489 | XM_021913548.1 | CqACO175 | 372 | 42276.55 | 5.99 | 41.47 | 87.04 | -0.4 | Cytoplasmic |
| LOC110734124 | XM_021914178.1 | CqACO176 | 341 | 39104.99 | 5.62 | 34.11 | 84.87 | -0.286 | Cytoplasmic |
| LOC110734125 | XM_021914179.1 | CqACO177 | 337 | 38266.8 | 5.53 | 43.55 | 88.52 | -0.294 | Cytoplasmic |
| LOC110734126 | XM_021914180.1 | CqACO178 | 370 | 41657.81 | 5.89 | 46.94 | 90.86 | -0.204 | Cytoplasmic |
| LOC110734126 | XM_021914181.1 | CqACO179 | 327 | 36918.43 | 6.19 | 43.77 | 88.81 | -0.248 | Cytoplasmic |
| LOC110734238 | XM_021914299.1 | CqACO180 | 326 | 36174.38 | 7.72 | 47.02 | 90.61 | -0.19 | Chloroplast |
| LOC110734370 | XM_021914504.1 | CqACO181 | 335 | 37842.07 | 5.36 | 45.41 | 83.97 | -0.367 | Cytoplasmic |
| LOC110734371 | XM_021914505.1 | CqACO182 | 341 | 39214.15 | 6.23 | 33.65 | 83.4 | -0.379 | Cytoplasmic |
| LOC110734378 | XM_021914513.1 | CqACO183 | 369 | 42284.31 | 6.29 | 50.97 | 86.18 | -0.435 | Cytoplasmic |
| LOC110734378 | XM_021914514.1 | CqACO184 | 365 | 41812.69 | 6.29 | 51.83 | 85.26 | -0.457 | Cytoplasmic |
| LOC110734507 | XM_021914672.1 | CqACO185 | 360 | 40917.17 | 6.43 | 37.98 | 90.39 | -0.255 | Cytoplasmic |
| LOC110734605 | XM_021914767.1 | CqACO186 | 337 | 38172.48 | 5.22 | 36.12 | 87.33 | -0.242 | Cytoplasmic |
| LOC110734688 | XM_021914829.1 | CqACO187 | 374 | 43250.5 | 7.14 | 59.05 | 77.89 | -0.516 | Nuclear |
| LOC110736027 | XM_021916185.1 | CqACO188 | 375 | 43395.28 | 6.32 | 34.91 | 67.33 | -0.497 | Cytoplasmic |
| LOC110736751 | XM_021917047.1 | CqACO189 | 329 | 36685.64 | 5.28 | 33.73 | 90.03 | -0.301 | Cytoplasmic |
| LOC110738199 | XM_021918570.1 | CqACO190 | 364 | 40854.49 | 5.65 | 29.86 | 84.04 | -0.316 | Cytoplasmic |
| LOC110738490 | XM_021918903.1 | CqACO191 | 374 | 43266.52 | 6.57 | 57.3 | 78.93 | -0.508 | Nuclear |
| LOC110739930 | XM_021920408.1 | CqACO192 | 375 | 42147.58 | 7.72 | 38.41 | 85.47 | -0.301 | Mitochondrial |

Table S3 Offboarding data statistics

| Sample | Reads No. | Bases (bp) | Q30 (bp) | N (%) | Q20 (%) | Q30 (%) |
| --- | --- | --- | --- | --- | --- | --- |
| LZR1 | 44516694 | 6722020794 | 6412316110 | 0.000646 | 98.57 | 95.39 |
| LZR2 | 42397458 | 6402016158 | 6063682256 | 0.000657 | 98.3 | 94.71 |
| LZR3 | 42139358 | 6363043058 | 6078825487 | 0.000648 | 98.61 | 95.53 |
| LZW1 | 45173678 | 6821225378 | 6467606506 | 0.001267 | 98.36 | 94.81 |
| LZW2 | 42334984 | 6392582584 | 6055669082 | 0.000646 | 98.28 | 94.72 |
| LZW3 | 46730134 | 7056250234 | 6696804109 | 0.000657 | 98.37 | 94.9 |
| ZR1 | 48847874 | 7376028974 | 6996348862 | 0.00065 | 98.35 | 94.85 |
| ZR2 | 42936226 | 6483370126 | 6166255952 | 0.000678 | 98.45 | 95.1 |
| ZR3 | 52806600 | 7973796600 | 7614093042 | 0.000651 | 98.6 | 95.48 |
| ZW1 | 44207716 | 6675365116 | 6286251366 | 0.001342 | 98.1 | 94.17 |
| ZW2 | 42752906 | 6455688806 | 6125080682 | 0.000654 | 98.35 | 94.87 |
| ZW3 | 44258974 | 6683105074 | 6341195593 | 0.000653 | 98.36 | 94.88 |

Note: Sample: the name of the sample;

Reads No.: total number of Reads;

Bases (bp): total number of bases;

Q30 (bp): total number of bases with base recognition accuracy above 99.9%;

N (%): percentage of ambiguous bases;

Q20 (%): percentage of bases with a base recognition accuracy of 99% or more;

Q30 (%): percentage of bases with base recognition accuracy of 99.9% or more

Table S4 Data filtering statistics

| Sample | Clean Reads No. | Clean Data (bp) | Clean Reads % | Clean Data % |
| --- | --- | --- | --- | --- |
| LZR1 | 42085126 | 6354854026 | 94.53 | 94.53 |
| LZR2 | 39989962 | 6038484262 | 94.32 | 94.32 |
| LZR3 | 39664794 | 5989383894 | 94.12 | 94.12 |
| LZW1 | 42661182 | 6441838482 | 94.43 | 94.43 |
| LZW2 | 39832392 | 6014691192 | 94.08 | 94.08 |
| LZW3 | 43961806 | 6638232706 | 94.07 | 94.07 |
| ZR1 | 45954480 | 6939126480 | 94.07 | 94.07 |
| ZR2 | 40455778 | 6108822478 | 94.22 | 94.22 |
| ZR3 | 49736400 | 7510196400 | 94.18 | 94.18 |
| ZW1 | 41697916 | 6296385316 | 94.32 | 94.32 |
| ZW2 | 40266080 | 6080178080 | 94.18 | 94.18 |
| ZW3 | 41714656 | 6298913056 | 94.25 | 94.25 |

Note: Sample: sample name;

Clean Reads No: number of high quality sequence reads;

Clean Data (bp): number of high quality sequence bases;

Clean Reads %: percentage of high quality sequence reads to sequencing reads;

Clean Data %: percentage of sequenced bases from high-quality sequences

Table S5 KEGG analysis of LZR_vs_ZR

| Pathway  ID | category | subcategory | Description | GeneRatio | pvalue | Gene  ID | Family gene |
| --- | --- | --- | --- | --- | --- | --- | --- |
| cqi00270 | Metabolism | Amino acid metabolism | Cysteine and methionine metabolism | 7/17 | 1.65E-08 | LOC110731284/LOC110681722/LOC110732609/LOC110686958/LOC110686006/LOC110720713/  LOC110728973 | CqACO156/CqACS1/CqACS34/CqACS4/CqSAMDC1/CqSAMDC5/  CqSPMS2 |
| cqi00330 | Metabolism | Amino acid metabolism | Arginine and proline metabolism | 6/17 | 1.82E-08 | LOC110705188/LOC110718863/LOC110701998/LOC110686006/LOC110720713/LOC110728973 | CqADC1/CqADC2/CqPAO10/CqSAMDC1/CqSAMDC5/CqSPMS2 |
| cqi00950 | Metabolism | Biosynthesis of other secondary metabolites | Isoquinoline alkaloid biosynthesis | 3/17 | 3.90E-05 | LOC110732609/LOC110686958/LOC110699680 | CqACS34/CqACS4/  CqCuAO4 |
| cqi00941 | Metabolism | Biosynthesis of other secondary metabolites | Flavonoid biosynthesis | 4/17 | 4.07E-05 | LOC110724781/LOC110725334/LOC110694697/  LOC110714529 | CqACO113/CqACO119/CqACO37/CqACO83 |
| cqi00360 | Metabolism | Amino acid metabolism | Phenylalanine metabolism | 3/17 | 0.0001409 | LOC110732609/LOC110686958/LOC110699680 | CqACS34/CqACS4/  CqCuAO4 |
| cqi00960 | Metabolism | Biosynthesis of other secondary metabolites | Tropane, piperidine and pyridine alkaloid biosynthesis | 3/17 | 0.0003413 | LOC110732609/LOC110686958/LOC110699680 | CqACS34/CqACS4/  CqCuAO4 |
| cqi00350 | Metabolism | Amino acid metabolism | Tyrosine metabolism | 3/17 | 0.0003560 | LOC110732609/LOC110686958/LOC110699680 | CqACS34/CqACS4/  CqCuAO4 |
| cqi00904 | Metabolism | Metabolism of terpenoids and polyketides | Diterpenoid biosynthesis | 2/17 | 0.0018263 | LOC110731393/  LOC110732989 | CqACO158/CqACO169 |
| cqi00130 | Metabolism | Metabolism of cofactors and vitamins | Ubiquinone and other terpenoid-quinone biosynthesis | 2/17 | 0.0080384 | LOC110732609/  LOC110686958 | CqACS34/CqACS4 |
| cqi00400 | Metabolism | Amino acid metabolism | Phenylalanine, tyrosine and tryptophan biosynthesis | 2/17 | 0.0085017 | LOC110732609/  LOC110686958 | CqACS34/CqACS4 |
| cqi00410 | Metabolism | Metabolism of other amino acids | beta-Alanine metabolism | 2/17 | 0.01207111 | LOC110699680/  LOC110701998 | CqCuAO4/CqPAO10 |

Table S6 The gene primer sequence information

| genes | Forward primer | Reverse primer |
| --- | --- | --- |
| CqADC2 | CCGTCAATCCATTTGGCGAC | GACCCAACCCACCATTCGAT |
| CqSPDS1 | GTCATCTCCCACTTTGCTCT | GAGCTACTTCTCGCAGGACA |
| CqSAMDC1 | ATGGCTGGAGACAACAACACT | ACAATGGTGCACTCAGCAGG |
| CqPAO10 | ATTTGGTGTCTCACTGGGGC | GGAAAACTAGCGCTTGTGGC |
| CqACS5 | CATTGCCGCTGATGCATACT | TCCATGAACTTCTTGTGGAATGC |
| CqACO59 | CAGTGATCTACCCTGCACCG | CTTGGCTCCTTAGGCTGGAA |


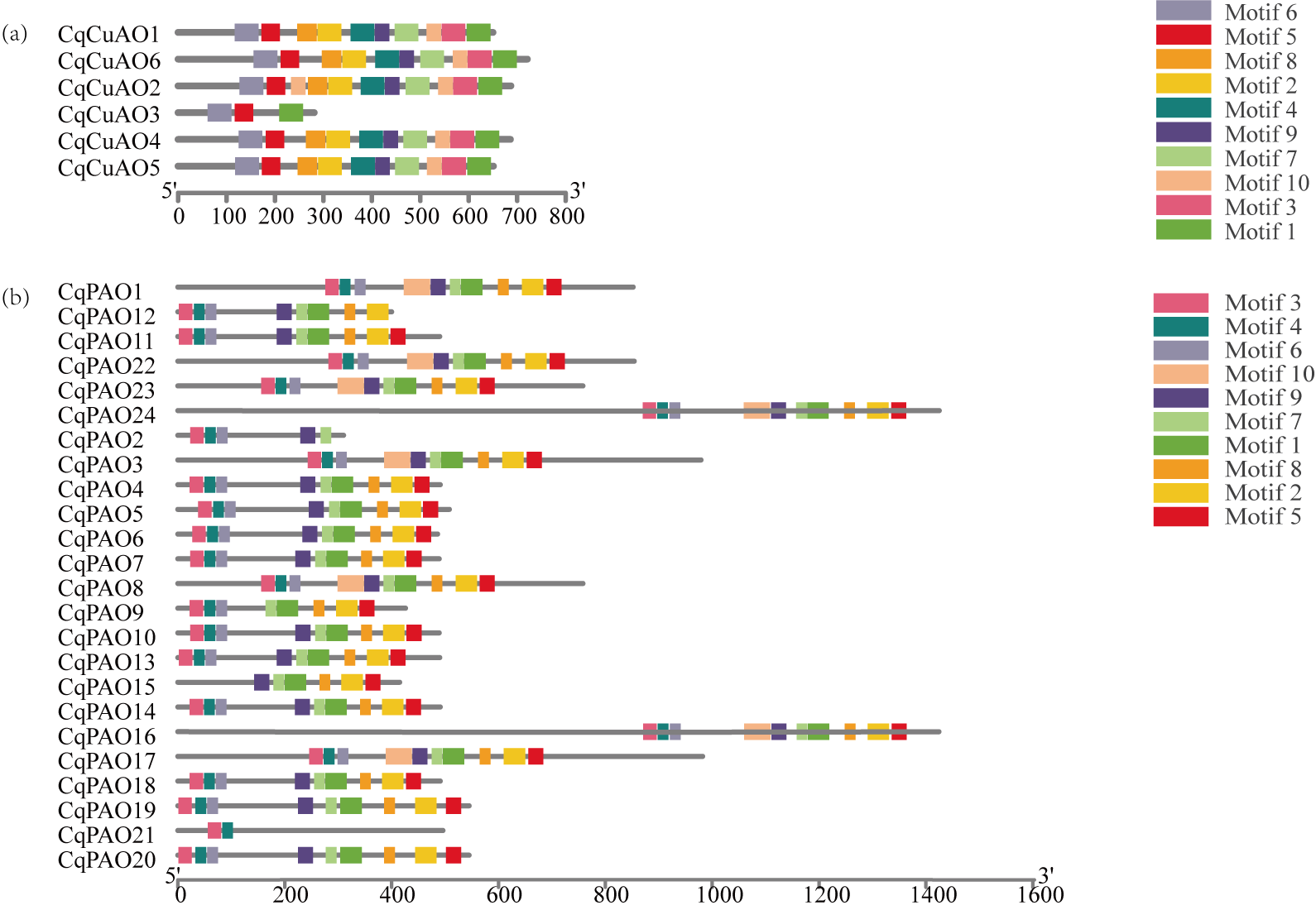


Figure S1 Conserved motifs of quinoa PA catabolic genes

(a) *CqCuAO* family, (b) *CqPAO* family


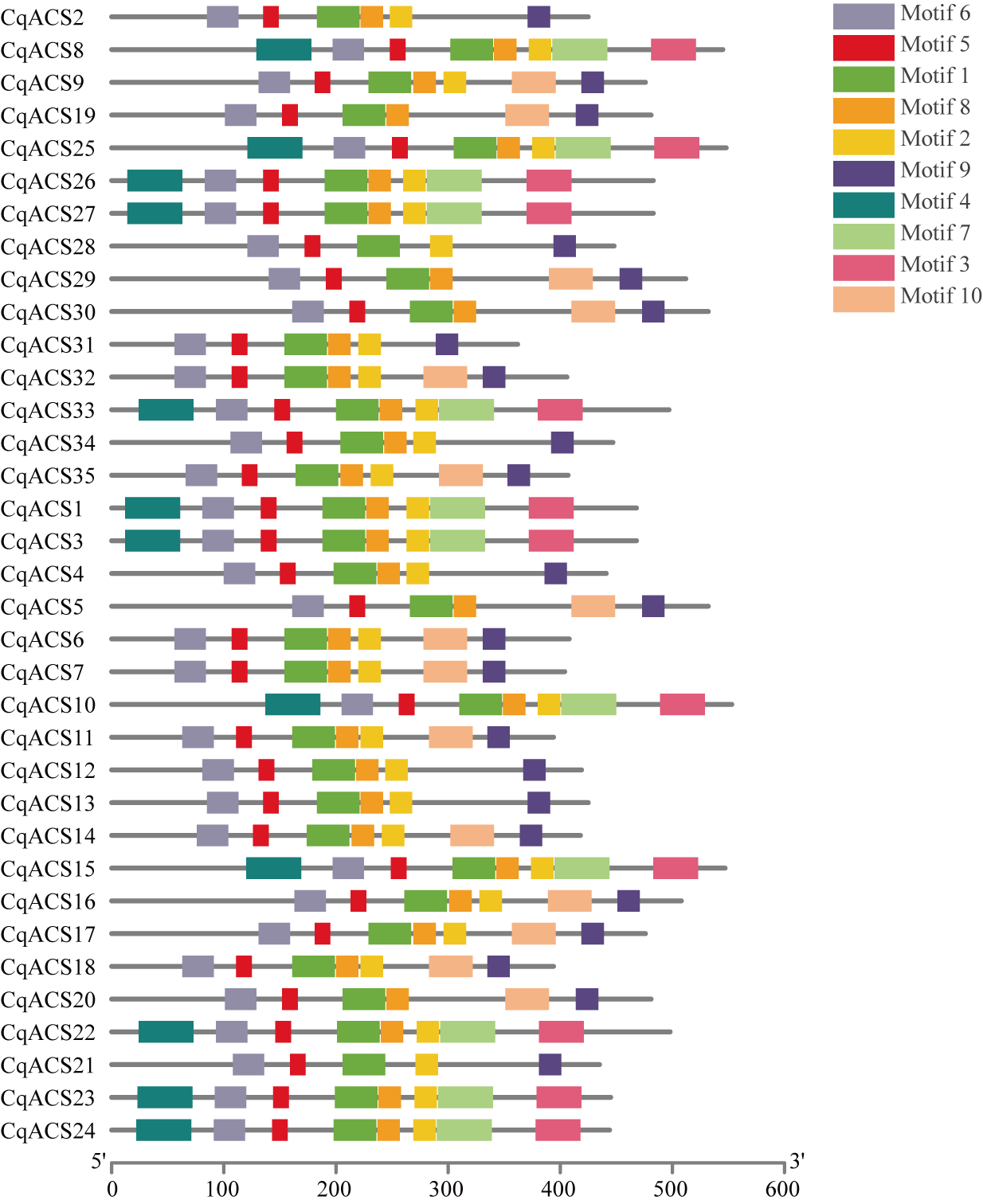


Figure S2 Conserved motifs of the *CqACS* family of quinoa ethylene synthesis genes

Figure S3 Conserved motifs of the *CqACO* family of quinoa ethylene synthesis genes


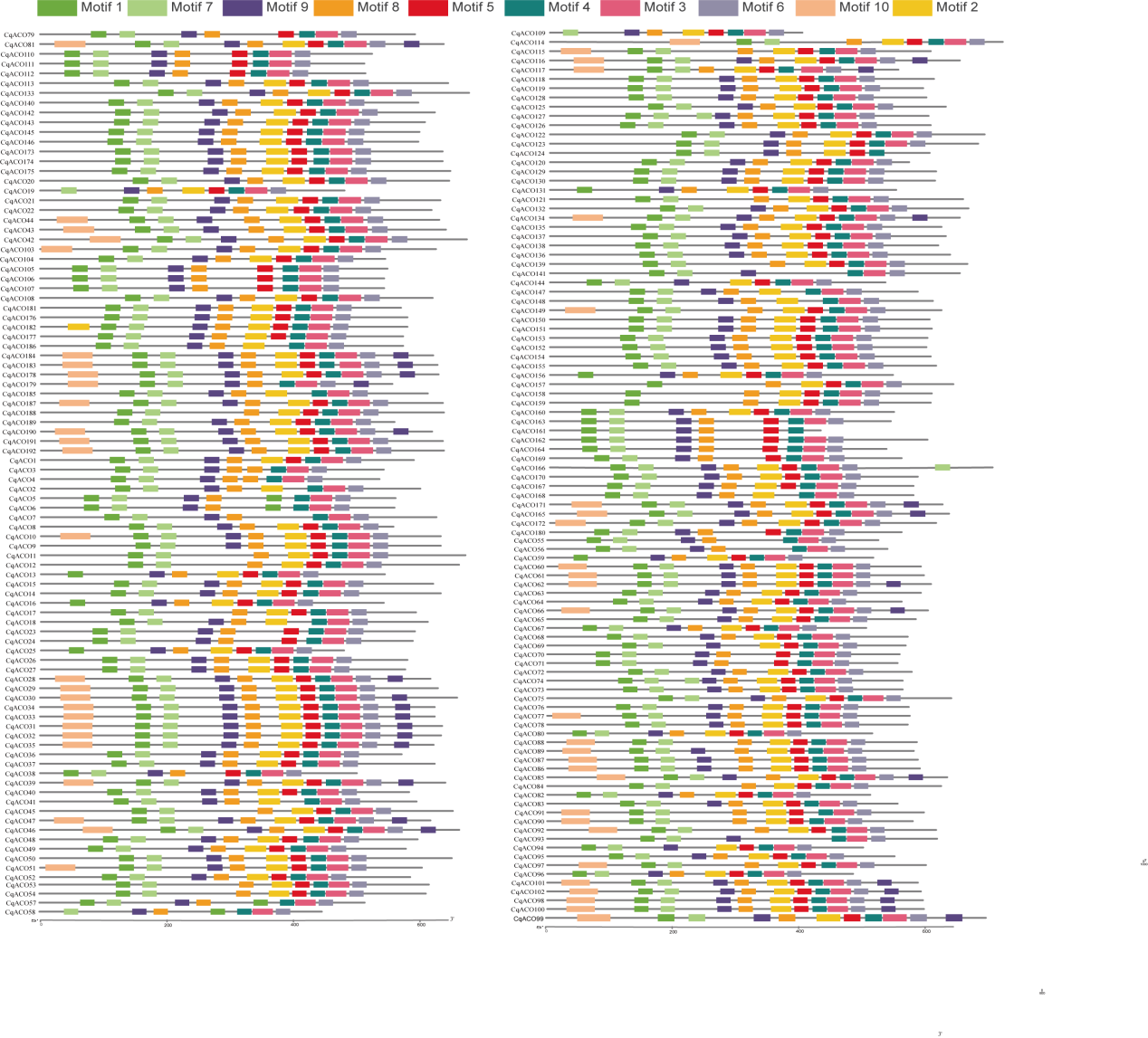

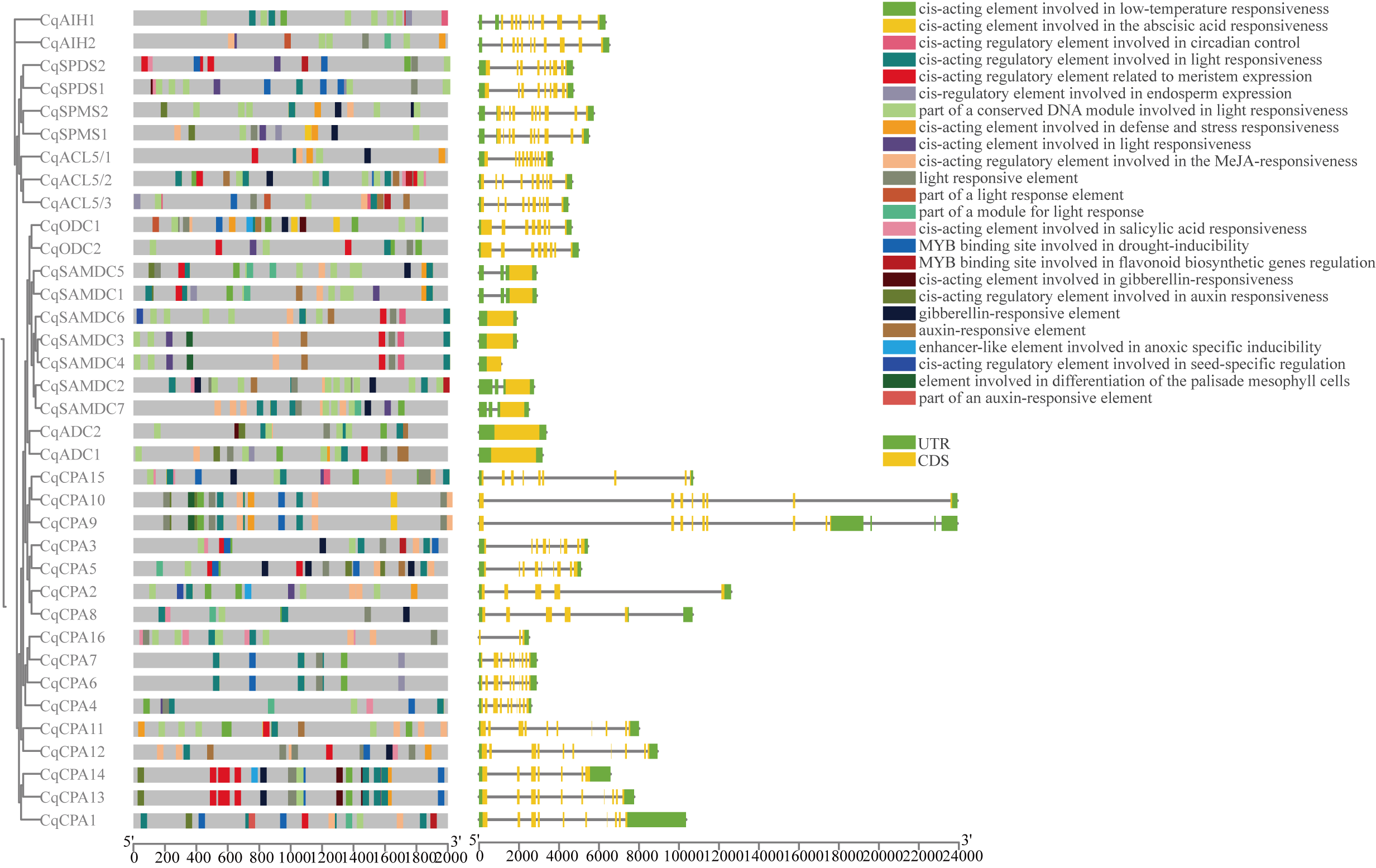


Figure S4 Analysis of promoter elements and gene structure of the PA synthetic gene family


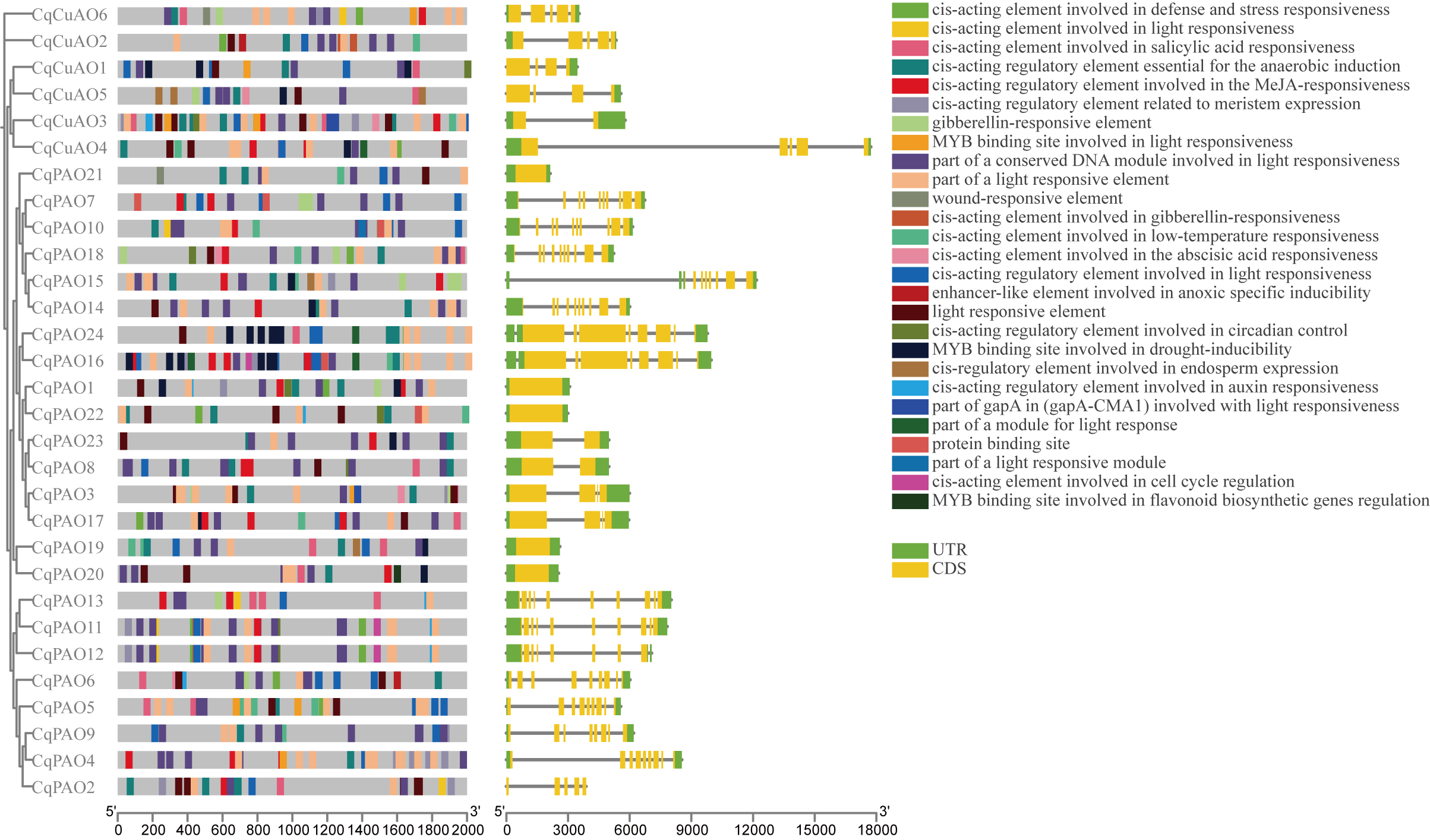


Figure S5 Analysis of promoter elements and gene structure of the PA catabolic family


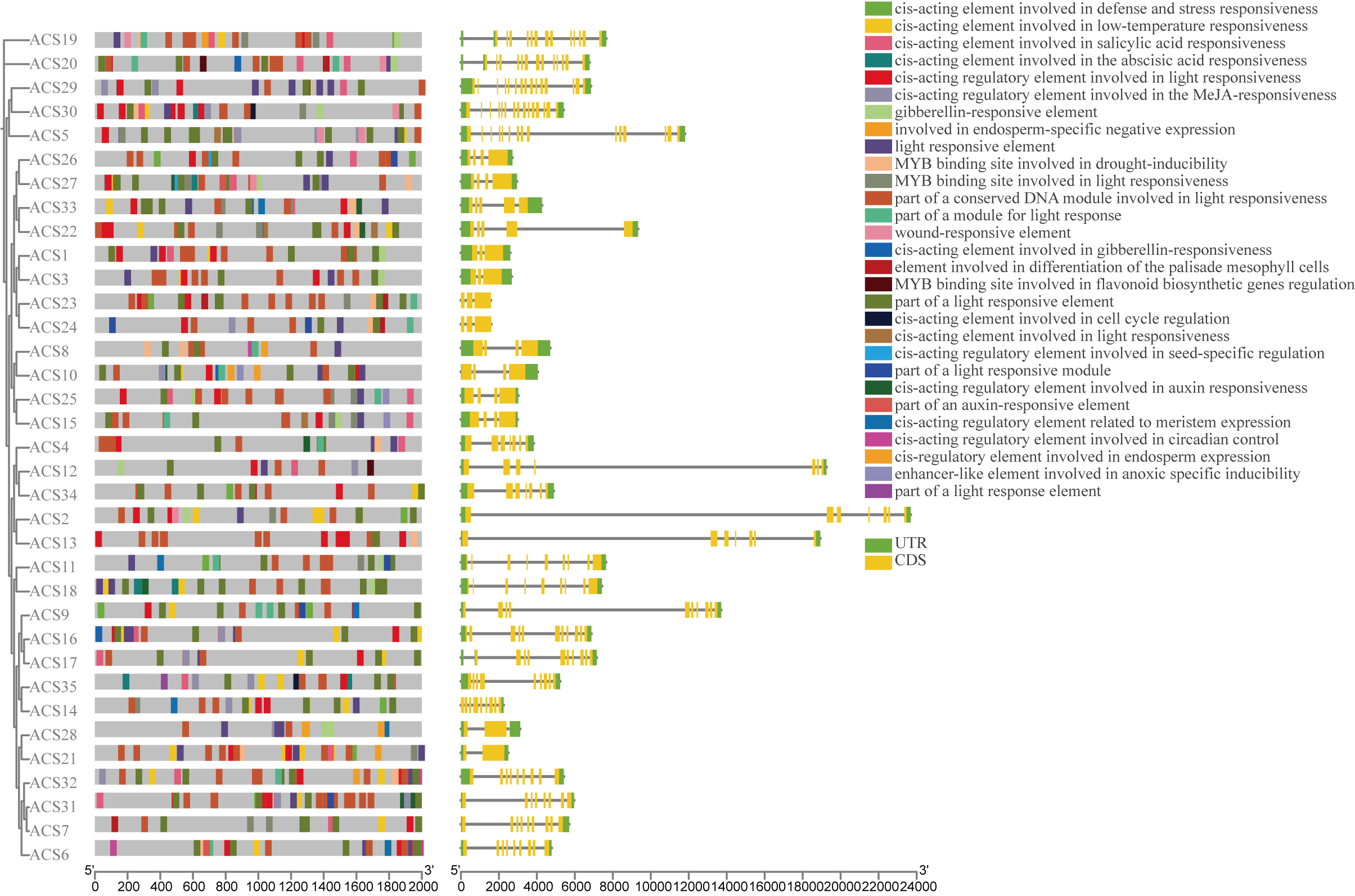


Figure S6 Analysis of promoter elements and gene structure of the CqACS family of ethylene synthesis genes


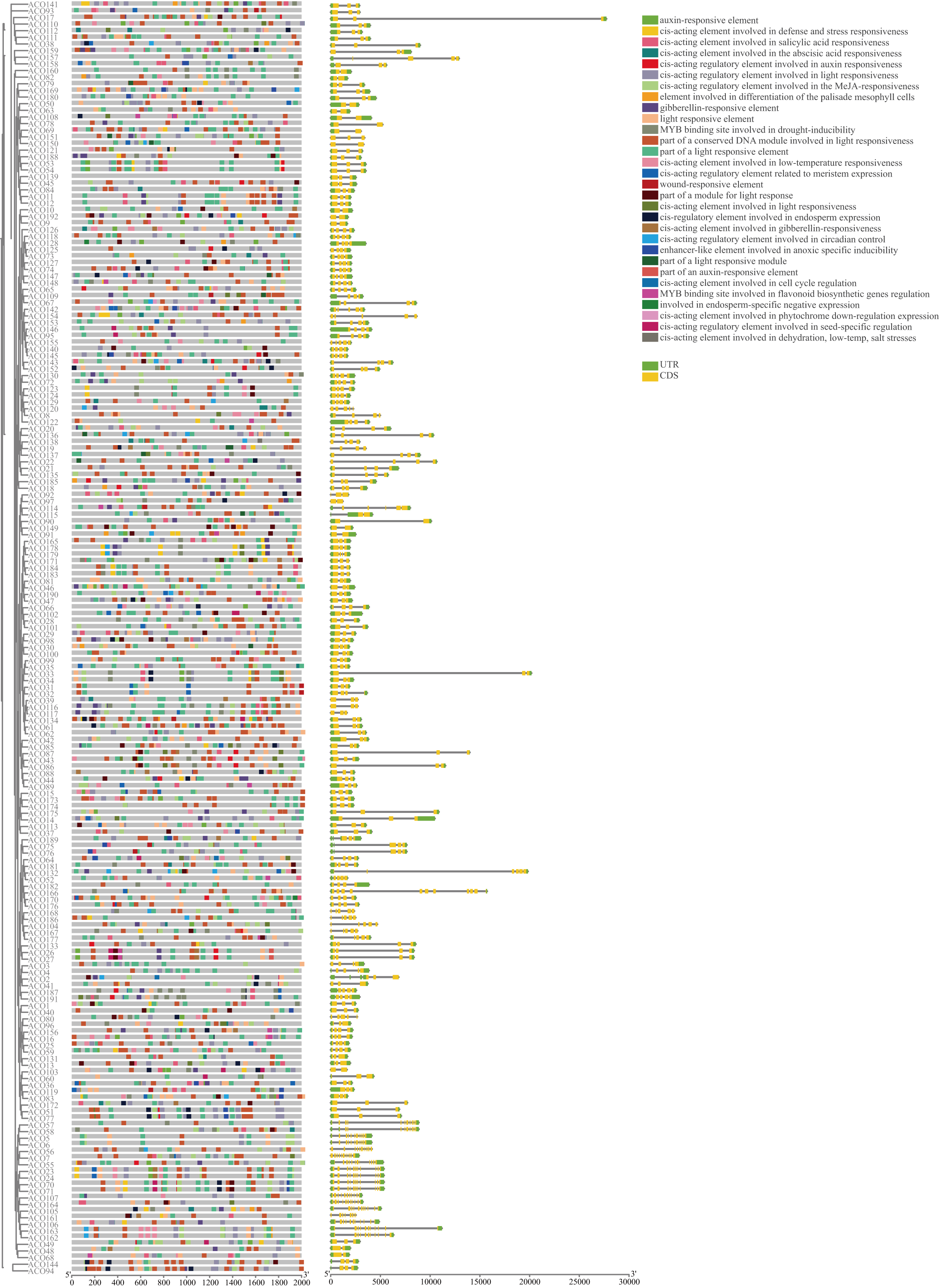


Figure S7 Analysis of promoter elements and gene structure of the CqACO family of ethylene synthesis genes


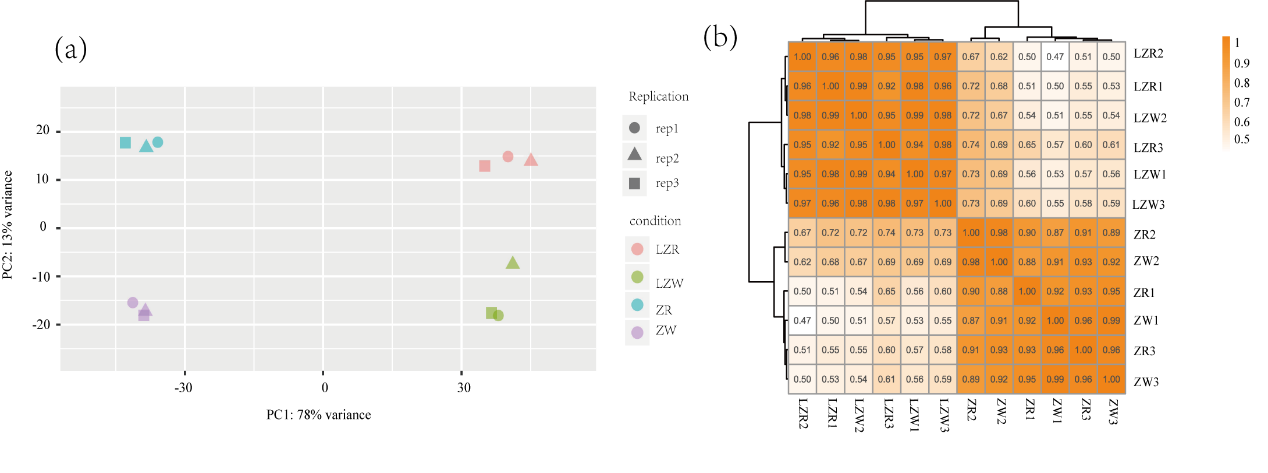


Figure S8 Sequencing quality testing

(a) Quinoa transcriptome PCA, (b) Analysis and sample correlation test


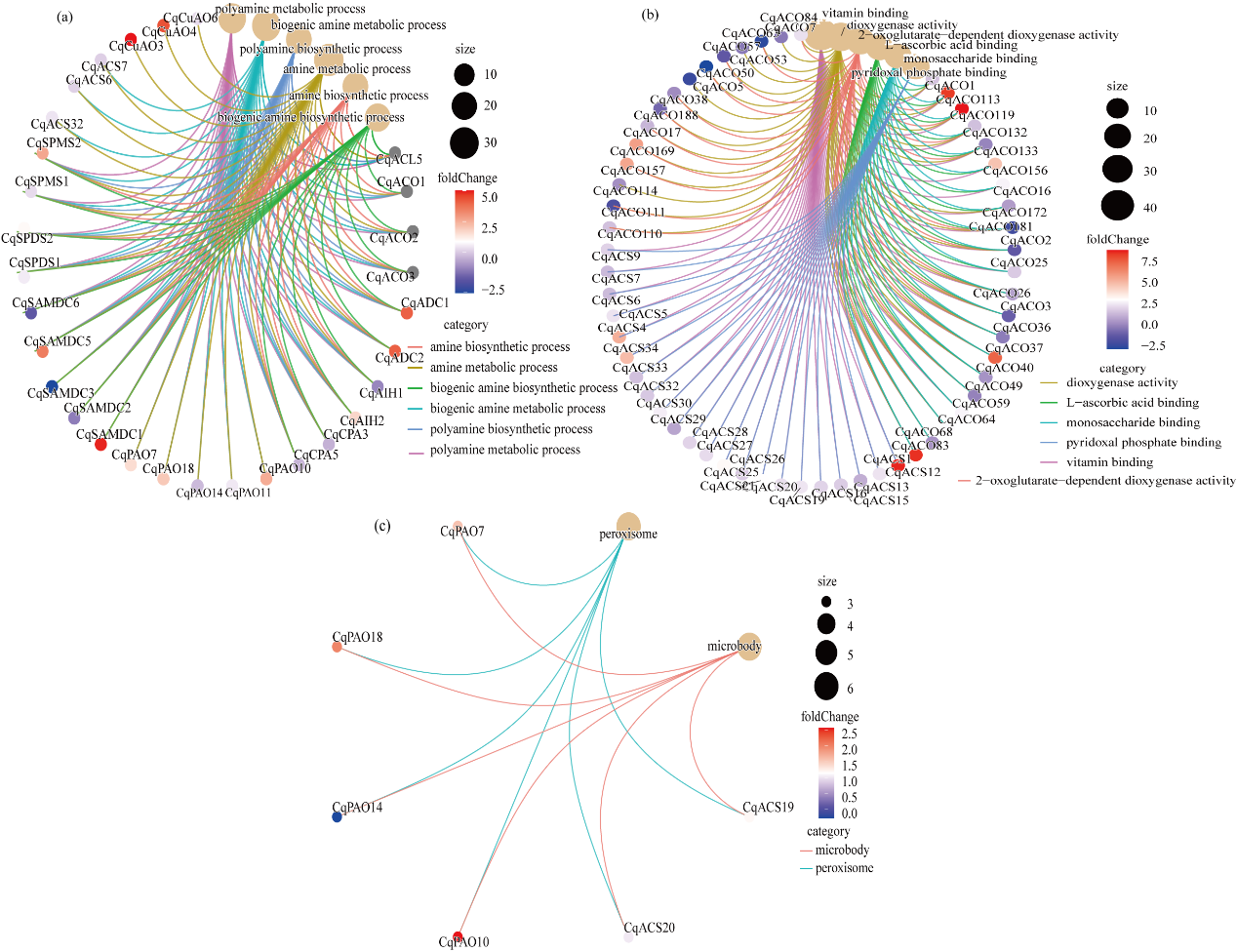


Figure S9. GO enrichment of genes in PA metabolism and ethylene synthesis,

1. Biological process, (b) Molecular function, (c) Cellular component


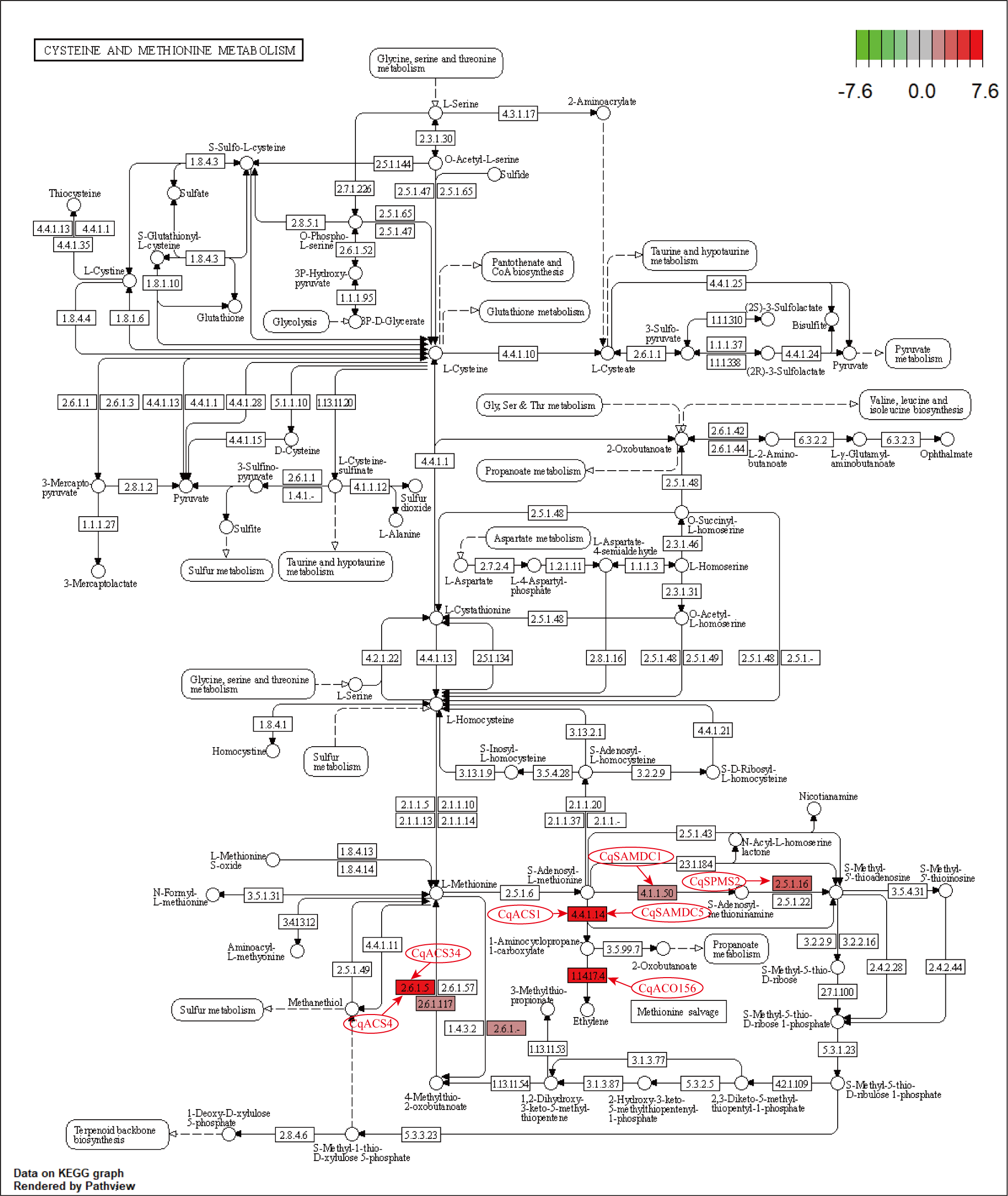


Figure S10 Cysteine and methionine metabolism pathway map
